# Highly-efficient RuNi single-atom alloy catalysts toward chemoselective hydrogenation of nitroarenes

**DOI:** 10.1038/s41467-022-30536-9

**Published:** 2022-06-08

**Authors:** Wei Liu, Haisong Feng, Yusen Yang, Yiming Niu, Lei Wang, Pan Yin, Song Hong, Bingsen Zhang, Xin Zhang, Min Wei

**Affiliations:** 1 State Key Laboratory of Chemical Resource Engineering, Beijing Advanced Innovation Center for Soft Matter Science and Engineering, Beijing University of Chemical Technology, Beijing, 100029 P. R. China; 2 Shenyang National Laboratory for Materials Science, Institute of Metal Research, Chinese Academy of Sciences, Shenyang, 110016 P. R. China

**Keywords:** Heterogeneous catalysis, Catalyst synthesis, Porous materials

## Abstract

The design and exploitation of high-performance catalysts have gained considerable attention in selective hydrogenation reactions, but remain a huge challenge. Herein, we report a RuNi single atom alloy (SAA) in which Ru single atoms are anchored onto Ni nanoparticle surface via Ru–Ni coordination accompanied with electron transfer from sub-surface Ni to Ru. The optimal catalyst 0.4% RuNi SAA exhibits simultaneously improved activity (TOF value: 4293 h^–1^) and chemoselectivity toward selective hydrogenation of 4-nitrostyrene to 4-aminostyrene (yield: >99%), which is, to the best of our knowledge, the highest level compared with reported heterogeneous catalysts. In situ experiments and theoretical calculations reveal that the Ru–Ni interfacial sites as intrinsic active centers facilitate the preferential cleavage of N–O bond with a decreased energy barrier by 0.28 eV. In addition, the Ru–Ni synergistic catalysis promotes the formation of intermediates (C_8_H_7_NO* and C_8_H_7_NOH*) and accelerates the rate-determining step (hydrogenation of C_8_H_7_NOH*).

## Abstract (web summary)

Catalytic hydrogenation of available nitroarenes is an environmentally benign and recyclable approach to synthesize value-added amines. Here, the authors design a RuNi single atom alloy with host-dopant synergistic effect, which exhibits outstanding performance toward nitroarenes hydrogenation.

## Introduction

Functionalized aromatic amines, as the vital building blocks of fine chemicals, have crucial applications in industry for the production of pharmaceuticals, agrochemicals, pigments, and polymers^1–3^. Catalytic hydrogenation of readily available nitroarenes over heterogeneous catalysts is an environmentally benign and recyclable approach to the synthesis of value-added amines, which have attracted considerable attention^4–7^. However, the chemoselective reduction of −NO_2_ group while reserving the other reducible groups (e.g., alkenes, alkynes, halogen, or ketones) is a highly desirable yet rather rough task^8–11^. Therefore, it is of great necessity to rationally exploit heterogeneous catalysts to exclusively reduce the −NO_2_ group without affecting the other reducible bonds^12–14^. Many non-precious metal catalysts have been explored to catalyze the nitroarenes hydrogenation, but the rigorous reaction conditions hinder their comprehensive applications^15,16^. Although precious metal catalysts (e.g., Ru^17^, Pt^18–20^, or Pd^21–23^) are active under mild reaction conditions, they generally suffer from high cost, dissatisfactory selectivity and low atomic utilization. Hence, how to acquire highly active and chemoselective catalysts via rational design and regulation on fine structure remains a big challenge^24–26^.

Single atom alloy (SAA) catalysts, with their maximized noble metal utilization efficiency, specific geometric, and electronic structure, provide unique active centers and result in outstanding catalytic properties that breaks the intrinsic linear scaling relationships^27–31^. The doped single atoms and host metal atoms in SAA can serve as dual-active sites which promote the activation of multiple substrates, or enable different elementary reaction steps occurring at different sites^32–34^. In addition, the heterometal combinations between isolated doped metal atoms and host metal may give rise to a special synergistic effect, although the understanding of this synergy of the two sets of metal sites at atomic scale still remain controversial^35–38^. As for the hydrogenation reaction of functionalize nitroarenes, the electronic and geometric structure of the active sites should be engineered to accelerate the exclusive activation of nitro-group. The electron-deficient nitro group tends to preferentially adsorb on the nucleophilic site on the surface of catalyst; whereas in the subsequent hydrogenation route, the cooperative catalysis of adjacent active center would play a vital role in governing the reactivity and chemoselectivity^4,25^. This evokes us to explore SAA catalyst as a promising candidate to improve catalytic performance toward chemoselective hydrogenation of nitroarenes, and further reveal the relationship between active site structure and catalytic property at atomic scale.

Herein, a RuNi single atom alloy catalyst was prepared based on a two-step synthesis method including a structural topological transformation of layered double hydroxides (LDHs) followed by a galvanic replacement treatment. The RuNi SAA exhibits extraordinary catalytic performance for chemoselective hydrogenation of 4-nitrostyrene to 4-aminostyrene (yield >99%), and the turnover frequency (TOF) value reaches up to ~4300 mol·mol_Ru_^−1^·h^−1^, which stands at the highest level among heterogeneous catalysts ever reported under analogous reaction conditions. Electron microscopic and spectroscopic characterizations show that isolated Ru atoms are dispersed on Ni nanoparticles (~8 nm) surface to form a stable Ru–Ni coordination, which results in the negative Ru sites (Ru^*δ*–^) due to electron transfer from sub-surface Ni to Ru. In situ FT-IR, XAFS investigations and density functional theory (DFT) calculations confirm that Ru–Ni interfacial sites as intrinsic active centers facilitate the activation adsorption of nitro-group via both Ru−O and Ni−O bonds with a lower energy barrier (0.46 eV), in contrast to monometallic Ni catalyst (0.74 eV). Moreover, hydrogen undergoes dissociation on adjacent Ni sites, followed by the hydrogenation of intermediates (C_8_H_7_NO* and C_8_H_7_NOH*) on the Ru^*δ*–^ sites. This host-dopant synergistic effect in RuNi SAA catalyst results in outstanding activity and selectivity toward nitroarenes hydrogenation, which can be extended to other rare precious metal catalysts used in structure-sensitive reactions.

## Results and discussion

### Synthesis and characterization of RuNi bimetallic catalysts

**Fig. 1:**
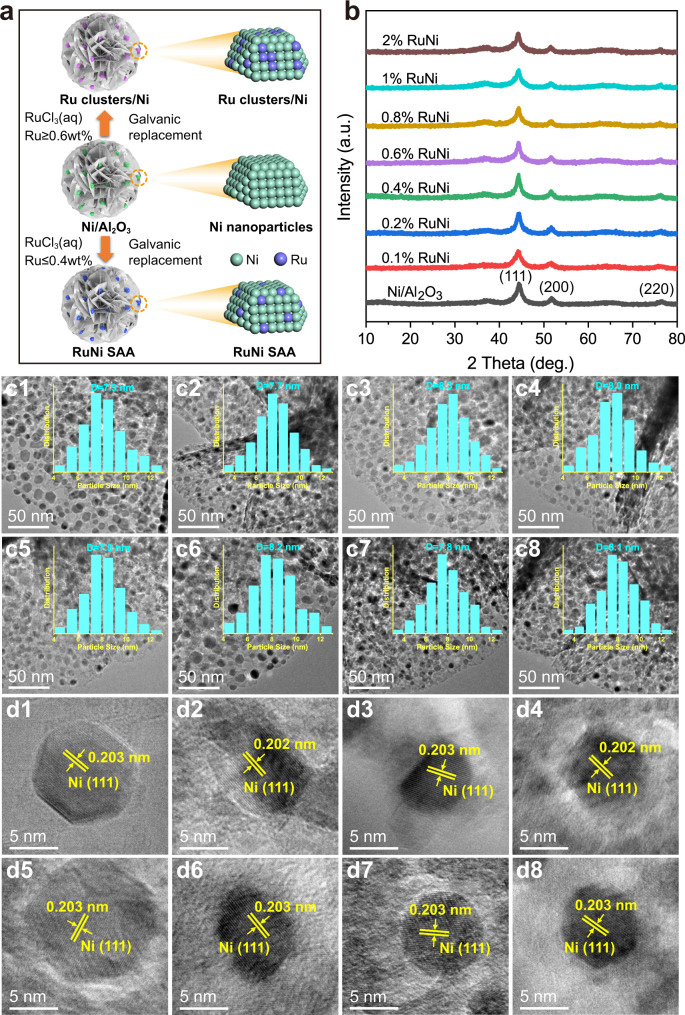
Preparation route, structure, and morphology characterizations of RuNi bimetallic samples. **a** Schematic illustration for the synthesis route of RuNi/Al_2_O_3_ samples; **b** XRD patterns of Ni/Al_2_O_3_ and various RuNi/Al_2_O_3_ samples. **c1**−**c8** TEM images with corresponding particle size distribution and **d1**−**d8** HRTEM lattice fringe images of monometallic Ni, 0.1 wt.%, 0.2 wt.%, 0.4 wt.%, 0.6 wt.%, 0.8 wt.%, 1 wt.%, and 2 wt.% RuNi, respectively. ‘a. u.’ denotes arbitrary units.

A monometallic Ni supported on amorphous Al_2_O_3_ substrate was prepared based on structural topotactic transformation of layered double hydroxides (LDHs) precursors. Afterwards, a set of RuNi/Al_2_O_3_ bimetallic samples with various Ru loading (0.1−2 wt.%) were precisely synthesized by a galvanic replacement method to deposit Ru atoms onto the surface of Ni nanoparticles (NPs) (Fig. 1a). Inductively coupled plasma atomic emission spectroscopy (ICP−AES) measurement explicitly gives the element composition of Ru and Ni in these samples (Supplementary Table 1), which is close to the theoretical loading of the feed. SEM images (Supplementary Fig. 1) and BET results (Supplementary Figs. 2–9 and Supplementary Table 1) clearly reveal that the morphology structure and specific surface area of RuNi/Al_2_O_3_ samples do not show obvious change during galvanic replacement process. The XRD patterns (Fig. 1b) show series of characteristic reflections at 2*θ* 44.3°, 51.6°, and 76.1°, indexed to the (111), (200), and (220) of a typical Ni (JCPDS 004–0850) phase. It is noted that the RuNi samples do not display metallic or oxidic Ru reflection, implying a high dispersion of Ru species. Transmission electron microscopy (TEM) measurements of monometallic Ni and RuNi samples (Fig. 1c1−c8) show that Ni nanoparticles are well dispersed and anchored onto the amorphous Al_2_O_3_ support with a close particle size (7.7−8.3 nm). HRTEM images (Fig. 1d1−d8) display a uniform lattice spacing of ~0.203 nm in both Ni and RuNi samples, corresponding to Ni(111) plane; yet the lattice fringes of Ru species are absent. This indicates that the Ru atoms are highly dispersed on the surface of samples, without influence on the lattice constants of Ni. Meanwhile, the 2 wt.% Ru/Al_2_O_3_ was synthesized as a control sample via deposition precipitation method, in which Ru clusters were uniformly distributed on the surface of Al_2_O_3_ support (Supplementary Figs. 10–12).

### Catalytic behavior for chemoselective hydrogenation of 4-nitrostyrene

**Fig. 2:**
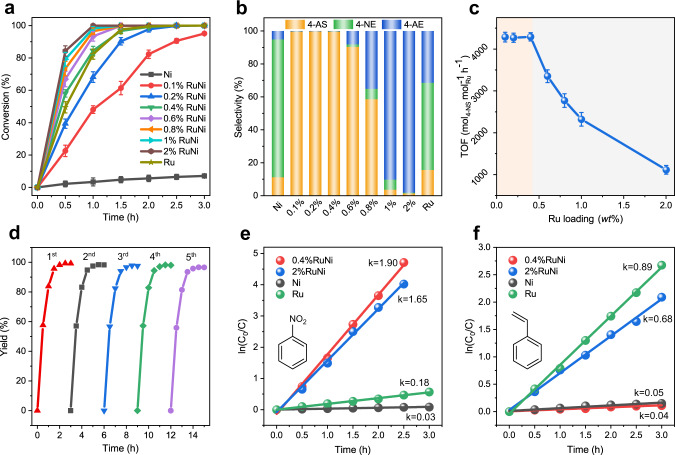
Catalytic performances of various samples toward nitroarenes hydrogenation. **a** Catalytic conversion of 4-nitrostyrene hydrogenation and **b** product distribution in the presence of monometallic Ni, Ru, and RuNi catalysts with various Ru loading (0.1−2 wt.%); **c** Turnover frequency (TOF) over RuNi catalysts as a function of per mole Ru under the catalytic dynamic range. **d** Reusability tests of 0.4 wt.% RuNi catalyst within five successive catalytic cycles. ln (*C*_0_/*C*) based on **e** nitrobenzene and **f** styrene vs. reaction time for the hydrogenation reaction of nitrobenzene and styrene mixture (1:1). Reaction conditions: 1 mmol of reactant; 8 ml of solvent (ethanol); 0.02 g of catalyst; 1 Mpa of H_2_, 60 °C, 3 h. Error bars were defined as the standard deviation by three replicate experiments.

The catalytic performance of RuNi samples were explored toward chemoselective hydrogenation of 4-nitrostyrene (4-NS) to 4-aminostyrene (4-AS). The conversion of 4-NS over pristine Al_2_O_3_ substrate is only 0.6% after 3 h (Supplementary Table 2), indicating that the catalytic effect of Al_2_O_3_ is negligible. As shown in Fig. 2a, the pristine Ni catalyst gives an extremely low catalytic activity with 7.1% 4-NS conversion after 3 h, whereas 100% conversion is achieved in the presence of monometallic Ru catalyst under the same conditions. In contrast to monometallic samples, all the RuNi catalysts show significantly enhanced hydrogenation activity (conversion: ~100%; 3 h), and the reaction rate is positively correlated with Ru content. This implies that the Ru species play a crucial role during the hydrogenation process. Interestingly, the product selectivity (Fig. 2b) is significantly diverse over various catalysts. For pristine Ni catalyst with a rather low activity, 4-nitroethylbenzene (4-NE) is the main product (selectivity: 83.6%) and the selectivity of 4-AS is 11.3%. In the case of monometallic Ru, the C=C bond in 4-NS is peculiarly prone to undergo hydrogenation than −NO_2_, resulting in the formation of 4-nitroethylbenzene (4-NE) or 4-aminoethylbenzene (4-AE); yet the selectivity of 4-AS is merely 15.7%. Surprisingly, RuNi catalysts with relatively low Ru loading (0.1−0.4 wt.%) exhibit an excellent selectivity (>99%) toward 4-aminostyrene (4-AS), indicating it is exclusively chemoselective for −NO_2_ rather than ethenyl. When Ru content is larger than 0.6 wt.%, the selectivity to 4-AS declines sharply whilst that of 4-AE augments instead, along with the increase of Ru loading. For the 2 wt.% RuNi catalyst, both nitro and ethenyl are highly hydrogenated with the selectivity toward 4-AE up to 98%. To investigate the influence of Ru dispersion state on the catalytic reaction, 0.4 wt.% Ru/Al_2_O_3_ sample was prepared (Supplementary Figs. 10, 13, and 14), whose Ru species was mainly dispersed as single atoms, accompanied with a small amount of Ru clusters (quasi-single atom Ru). The catalytic performance (Supplementary Table 2) shows that 0.4 wt.% Ru/Al_2_O_3_ gives an improved selectivity of 4-AS (67.5%) relative to 2 wt.% Ru/Al_2_O_3_ sample, but the activity is rather low (conversion: 12.9%; 3 h). Based on total surface metal sites determined by CO pulse chemisorption measurements, the turnover frequency (TOF_metal_) of RuNi catalysts was obtained under a low 4-NS conversion (Supplementary Fig. 15), which showed a trend of increase first and then decrease along with the increase of Ru loading (Supplementary Fig. 16). This indicates that not all the surface metal sites serve as the intrinsic active centers in RuNi catalysts. In addition, the TOF of RuNi catalysts was calculated based on Ru sites, to further reveal the intrinsic catalytic activity (Fig. 2c). With the increase of Ru loading from 0.1 wt.% to 0.4 wt.%, the RuNi catalysts exhibit an almost constant TOF value (4271−4293 h^−1^), indicating that the Ru species is located as atomic dispersion (might form RuNi SAA) and serves as the main active sites. However, the TOF value decreases significantly with a further increase of Ru loading (within 0.6−2 wt.%), which implies a change of intrinsic active site structure (from atomic dispersion to Ru nanoclusters). Moreover, the TOF of 0.4 wt.% RuNi (SAA) catalyst, to the best of our knowledge, stands at the highest level among metal catalysts reported previously under similar reaction conditions (Supplementary Table 3), which further demonstrates RuNi single atom alloy affords superior catalytic performance. Supplementary Fig. 17 shows catalytic performance of 0.4 wt.% RuNi (SAA) catalyst at various H_2_ pressure and temperature, from which 1 MPa of H_2_ pressure and 60 °C of reaction temperature are adopted as the optimal reaction parameters. The reusability of 0.4 wt.% RuNi sample was further evaluated (Fig. 2d), and no significant shrink in both activity and yield was observed within five successive recycles. The XRD pattern and TEM images (Supplementary Figs. 18 and 19) of the used 0.4 wt.% RuNi catalyst after 5 cycles do not show obvious change in crystal structure, indicating a high stability in the selective hydrogenation reaction. In addition, the 0.4 wt.% RuNi (SAA) catalyst also achieved excellent amines yield toward the chemoselective hydrogenation of other nitroarenes contained halogen, aldehyde, and hydroxyl group (Supplementary Table 4), which demonstrates its good applicability.

To further study the remarkable chemoselectivity difference, the hydrogenation of styrene and nitrobenzene mixture (1:1) was also performed in the presence of monometallic Ni, Ru, 0.4 wt.% RuNi, and 2 wt.% RuNi catalysts, respectively (Supplementary Fig. 20). Although the chemoselectivity for hydrogenation reaction of functional groups is consistent, some differences do exist in the selectivity of intramolecular and intermolecular hydrogenation, as a result of the molecular allosteric effect. As shown in Fig. 2e, f, the curve of ln (*C*_0_/*C*) as a function of reaction time give a straight line starting with the origin point, indicating a pseudo first-order reaction with respect to both nitrobenzene and styrene. Monometallic Ni catalyst shows extremely low hydrogenation rate constant toward both nitrobenzene (0.03 h^−1^) and styrene (0.05 h^−1^). Notably, a preferential styrene hydrogenation activity (rate constant: 0.89 h^−1^) is achieved over monometallic Ru catalyst, which is greatly larger than nitrobenzene hydrogenation (rate constant: 0.18 h^−1^). In the case of 0.4 wt.% RuNi (SAA) catalyst, nitrobenzene hydrogenation is more dynamically favorable than styrene hydrogenation (rate constant: 1.90 h^−1^ vs. 0.04 h^−1^), indicating a preferred hydrogenation of −NO_2_ group rather than C=C bond. For 2 wt.% RuNi catalysts, the rate constant of nitrobenzene hydrogenation (1.65 h^−1^) drops compared with that of 0.4 wt.% RuNi (but remains higher than monometallic catalysts), while the hydrogenation rate of styrene increases sharply (rate constant: 0.68 h^−1^). This further implies a significantly promoted catalytic activity and chemoselectivity toward −NO_2_ group over RuNi SAA with the synergistic effect between Ni and Ru.

### Investigations on structure-selectivity correlations

**Fig. 3:**
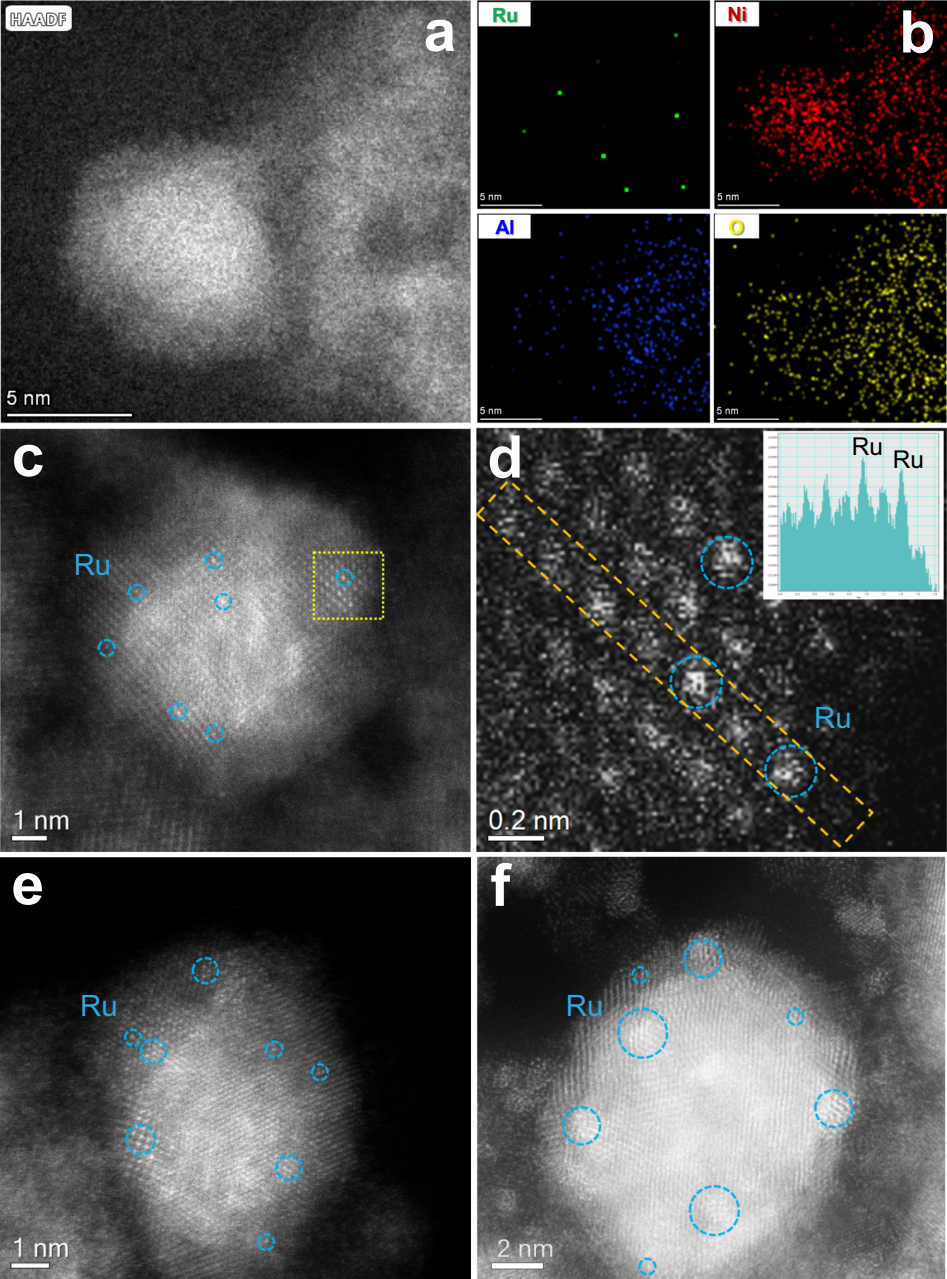
Electron micrology studies on RuNi catalysts. **a** HAADF–STEM image, **b** corresponding EDS mapping images, **c** high-resolution AC–HAADF–STEM image, and **d** enlarged STEM image and corresponding intensity profile of 0.4 wt.% RuNi sample. **e**, **f** AC–HAADF–STEM images of the 0.6 wt.% RuNi and 2 wt.% RuNi samples, respectively.

Aberration-correction high-angle annular dark-field scanning transmission electron microscopy (AC–HAADF–STEM) imaging technique and energy-dispersive spectroscopy (EDS) elemental mapping were conducted to intuitively ascertain the dispersion state of Ru and Ni species. EDS elemental mapping of 0.4 wt.% RuNi sample (Fig. 3a, b) illustrates a highly uniform dispersion of Ru on Ni nanoparticles rather than Al_2_O_3_ substrate; corresponding AC–HAADF–STEM image (Fig. 3c) shows that a number of atom-sized bright spots (highlighted by the blue arrows) attributed to individual Ru atoms are distributed on the surface of Ni NPs, without observation of Ru clusters or nanoparticles. Enlarged STEM image along with the intensity profile further verifies that Ru atoms are atomically dispersed on Ni NPs (Fig. 3d), demonstrating the formation of RuNi single atom alloy. For the 0.6 wt.% RuNi sample (Fig. 3e), both Ru single atoms and few Ru ensembles are observed on Ni NPs, suggesting a slight aggregation of Ru atoms due to the increase in loading. In the case of 2 wt.% RuNi sample, a number of large Ru clusters are detected on Ni NPs in HAADF–STEM image (Fig. 3f) and EDS elemental mapping (Supplementary Fig. 21), indicating a considerable aggregation of Ru.

**Fig. 4:**
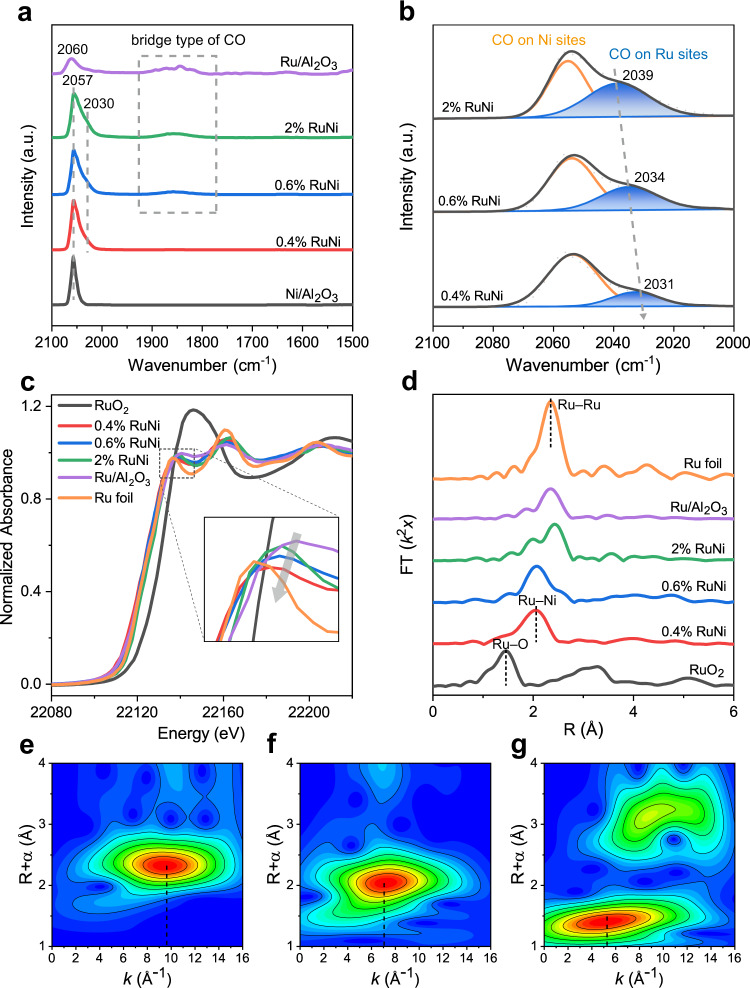
Fine-structure characterizations of RuNi catalysts. **a** In situ CO–DRIFTS spectra of Ni/Al_2_O_3_, Ru/Al_2_O_3_, and 0.4 wt.%, 0.6 wt.%, 2 wt.% RuNi samples, within 2100−1500 cm^−1^ by flowing He gas for 20 min. **b** The enlarged and Gaussian fitting spectra with a fixed peak position and FWHM of RuNi/Al_2_O_3_ samples. **c** In situ Ru K-edge XANES spectra and **d** EXAFS Fourier–transform spectra of various samples. *k*^2^-weighted wavelet transforms for the Ru K-edge XAFS signals of **e** Ru foil, **f** 0.4 wt.% RuNi, and **g** RuO_2_ sample based on Morlet wavelets. ‘a. u.’ denotes arbitrary units.

In situ DRIFTS spectra of CO adsorption (Fig. 4a) was performed to further explore structural details of 0.4 wt.%, 0.6 wt.%, and 2 wt.% RuNi samples, in comparison with Ni/Al_2_O_3_ and Ru/Al_2_O_3_ samples. CO adsorption on Ru/Al_2_O_3_ sample produces dominant peak at 2060 cm^–1^ and another broad peak at 1849 cm^–1^, which are assigned to linearly adsorbed CO at Ru and bridged-bonded CO at two contiguous Ru atoms, respectively^39,40^. For monometallic Ni sample, only a strong peak at 2057 cm^–1^ is observed, which is attributed to the linear CO on Ni site^41,42^. In the case of RuNi samples, in addition to the main peak at 2056 cm^–1^, an obvious shoulder peak centered at ~2030 cm^–1^ is observed. By using Gaussian peak fitting method, the profiles of RuNi samples within 2000−2100 cm^–1^ are reasonably deconvoluted and fitted to two peaks including CO linear-type adsorption on Ni site (2056 cm^–1^) and Ru site (2031−2039 cm^–1^) (Fig. 4b). Interestingly, a remarkable red shift of the linear-bonded CO peak on Ru site occurs from Ru/Al_2_O_3_ (2060 cm^–1^) to RuNi samples (2031–2039 cm^–1^), and the red shift gradually became pronounced with the decrease in Ru content. This indicates an enhanced electronegativity of Ru species in RuNi samples, as a result of electron transfer from Ni to Ru, increases the *d*-*π* electron feedback from Ru to CO 2π* antibonding orbital. Furthermore, for the 0.4 wt.% RuNi samples, no bridge adsorption peak is observed, implying that Ru species exists as isolated atoms (SAA) by Ni. In the case of 0.6 wt.% RuNi and 2 wt.% RuNi samples, the appearance of bridge-bonded CO confirms the existence of Ru multimer or clusters, which accords well with the AC–HAADF–STEM results.

The normalized Ru K-edge in situ X-ray absorption near-edge structure (XANES) spectra were conducted to study the electronic and geometric structure of RuNi samples in contrast to Ru foil and RuO_2_ samples. As shown in Fig. 4c, the intensity of white line shrinks gradually from Ru/Al_2_O_3_ to RuNi samples along with the decrease of Ru loading. Simultaneously, the white line intensity of Ni K-edge XANES spectra shows a slight increase from pristine Ni to RuNi samples (Supplementary Fig. 22). This indicates the changes in electron density and coordination environment of Ru species^43^. As shown in X-ray photoelectron spectroscopy (XPS) spectra (Supplementary Fig. 23), compared with the monometallic Ru and Ni, the Ru^0^ peak of RuNi samples shifts to lower binding energy whilst the Ni^0^ peak moves to higher binding energy, which further demonstrates the electron transfer from Ni atoms to Ru atoms in RuNi SAA. The Bader charge analysis of RuNi SAA(111) surface reveals that the isolated Ru atom carries negative charges (Ru^*δ*–^) transferred from sub-surface Ni atoms (Supplementary Fig. 24), which is in accordance with the in situ DRIFTS and XPS results. The Fourier transforms of the extended X-ray absorption fine spectrum (EXAFS) in the R space was performed to investigate the detailed coordination structure of Ru (Fig. 4d). The 0.4 wt.% RuNi sample exhibits a sharp peak located at ~2.1 Å, which is in the region between Ru–O shell (1.5 Å) and Ru–Ru shell (2.4 Å) and can be assigned to the Ru–Ni coordination^44,45^. The EXAFS data-fitting results (Supplementary Table 5 and Supplementary Figs. 25–28) manifest that the coordination number (CN) of Ru–Ni path is 5.4, while the Ru–Ru and Ru–O coordination are absent in 0.4 wt.% RuNi sample. This verifies that predominant Ru atoms are atomically dispersed and surrounded by Ni to form single atom alloy. Notably, the peak intensity of Ru–Ru coordination (~2.4 Å) arises in 0.6 wt.% RuNi sample and enhances in 2 wt.% RuNi sample. Exactly, the EXAFS curve fittings reveal that the Ru–Ru coordination number distinctly increases from 0 (0.4 wt.% RuNi) to 2.2 (0.6 wt.% RuNi) and further to 6.7 (2 wt.% RuNi), respectively, indicating the Ru atoms aggregate gradually upon increasing Ru loading. The *k*^2^-weighted wavelet transform (WT) for the Ru K-edge XAFS signals were further employed to study coordination environment of Ru species. As shown in Fig. 4e, the lobe of Ru foil at 2.3 Å, 9.7 Å^−1^ is ascribed to Ru–Ru contribution. The absence of lobes at *k* = 9.7 Å^−1^ and 5.3 Å^−1^ in 0.4 wt.% RuNi sample (Fig. 4f) excludes the central Ru bonds to Ru atom and O atom (Fig. 4g); meanwhile, a new lobe attributed to Ru–Ni contribution is observed at 2.1 Å, 7.1 Å^−1^, demonstrating the formation of SAA. Moreover, Ni K-edge EXAFS spectrum of different samples does not show significant differences (Supplementary Fig. 29), indicating that the coordination structure of Ni is less affected by the surface Ru atoms. In brief, the results of AC–HAADF–STEM, in situ CO–DRIFTS as well as in situ XAFS experiments substantiate the successful preparation of RuNi SAA catalyst, as well as the evolution of Ru species on Ni NPs from single atom to Ru multimers by increasing Ru loading. In addition, the HAADF–STEM image (Supplementary Fig. 30) and EXAFS spectrum (Supplementary Fig. 31) of the used RuNi SAA catalyst display that the dispersion state and coordination structure of Ru atoms do not show obvious change after 5 cycles, demonstrating a high stability of RuNi SAA catalyst.

### Mechanism insight of hydrogenation of 4-NS on RuNi SAA

H_2_-TPD measurement was performed to explore dissociated adsorption of hydrogen on various catalysts, and the results showed that all these catalysts give strong H_2_ dissociation ability with desorption peaks at ~100 °C (Supplementary Fig. 32). The quantitative analysis results (Supplementary Fig. 33) do not show obvious linear correlation between reaction activity and the amount of hydrogen desorption. Moreover, we carried out D_2_ isotopic experiments and a kinetic isotope effect (KIE) value of 1.31 (TOF_H_/TOF_D_) was obtained (Supplementary Fig. 34), indicating that the activation and dissociation of H_2_ are essential but not the rate-limiting step. DFT calculation was carried out to further study the adsorption and dissociation behavior of hydrogen on RuNi SAA in comparison with monometallic Ni (Supplementary Fig. 35). For RuNi SAA sample, H_2_ molecule preferentially undergoes chemical adsorption at the top site of Ru single atom with an adsorption energy of −0.76 eV. Subsequently, the hydrogen dissociates into two H active atoms at Ru–Ni hollow site of RuNi SAA overcoming an energy barrier of 0.02 eV. In addition to the Ru site, H_2_ molecule can also be chemically adsorbs on the top site of Ni atom adjacent to Ru (adsorption energy: −0.38 eV), and then dissociates into two H atoms at Ru–Ni and Ni–Ni hollow sites with a barrier of 0.06 eV. In contrast, the adsorption energy and dissociation energy barrier of H_2_ molecule on Ni(111) surface are −0.40 eV and 0.09 eV, respectively. The extremely low energy barriers and subtle difference indicate that H_2_ is prone to dissociate on both Ni and RuNi SAA surface (either Ni site or Ru site), which is not the key factor influencing their catalytic performance.

**Fig. 5:**
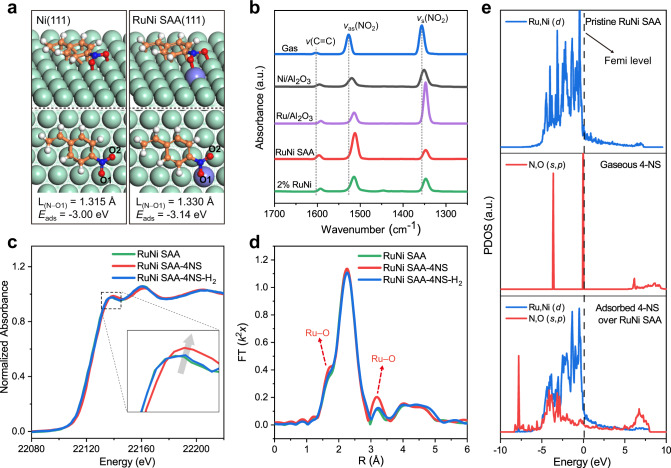
Identification of catalytic active sites. **a** Adsorption configurations (side and top views) of 4-NS on Ni(111) and RuNi SAA(111) surface, with the labeled bond length of N−O1 (L_(N−O1)_) and adsorption energies of 4-NS (*E*_ads_). Ru, violet; Ni, green; C, orange; O, red; N, blue; H, white. **b** In situ FT-IR spectra of gaseous and chemically adsorbed 4-NS on monometallic Ni, Ru, RuNi SAA (0.4 wt.%) and 2 wt.% RuNi, respectively. **c** In situ normalized XANES and **d** phase correction EXAFS Fourier–transform spectra at Ru K–edge of 0.4 wt.% RuNi SAA under 4-NS adsorption (RuNi SAA–4NS), and hydrogenation reaction stages (RuNi SAA–4NS–H_2_). **e** Projected density of states (PDOS) for pristine RuNi SAA(111) surface, N–O1 in gaseous 4-NS, and adsorbed 4-NS over RuNi SAA(111). ‘a. u.’ denotes arbitrary units.

The activation adsorption of specific functional group is of vital significant to selective hydrogenation of substrates. Hence, we conducted DFT calculations to investigate the possible adsorption configurations and active sites of 4-NS on RuNi SAA(111) surface, and the optimized results were shown in Supplementary Fig. 36. Clearly, the parallel configuration (Fig. 5a and Supplementary Fig. 36e), in which N atom is located at the Ru–Ni hollow site with two O atoms attaching to the Ru–Ni interface, displays the lowest level of adsorption energy (−3.14 eV). This indicates a more thermodynamically favorable adsorption mode in comparison with vertical and other parallel configurations (Supplementary Fig. 36a–d). Moreover, after the adsorption of 4-NS on the RuNi SAA(111), the bond length of N–O1 (L_(N−O1)_) in nitro-group is extended to 1.330 Å (Fig. 5a), which is significantly longer than that of gaseous 4-NS molecule (1.244 Å) (Supplementary Fig. 37) and even exceeds the L_(N−O1)_ on Ni(111) (1.315 Å). This suggests a prominently enhanced activation adsorption of N–O1 bond onto RuNi SAA surface, in comparison with the pristine Ni(111).

To further verify the adsorption behavior of 4-NS, in situ FT-IR measurements were performed over monometallic Ni, monometallic Ru, 0.4 wt.% RuNi (SAA), and 2 wt.% RuNi catalysts (Fig. 5b). The FT-IR spectra of gaseous 4-NS shows three characteristic peaks located at 1603, 1528, and 1356 cm^–1^, attributing to *ν*(C=C), *ν*_as_(NO_2_), and *ν*_s_(NO_2_), respectively^46–48^. In the presence of monometallic Ni, a red-shift of all these three bands is observed: *v*(C=C) (1595 cm^–1^), *ν*_as_(NO_2_) (1520 cm^–1^), and *ν*_s_(NO_2_) (1351 cm^–1^), indicating both C=C and −NO_2_ groups undergo chemisorption on the surface of Ni (most likely parallel adsorption configuration). For monometallic Ru sample, a more pronounced red-shift of these three bands is found (1591, 1514 and 1348 cm^–1^, respectively) relative to monometallic Ni, indicating a slightly enhanced adsorption of nitro-group and C=C bond on Ru. In the case of 0.4 wt.% RuNi (SAA), the *ν*(C=C) band is centered at 1596 cm^–1^, rather close to monometallic Ni (1595 cm^–1^), indicating vinyl tends to adsorb at the Ni sites of RuNi SAA. In addition, in striking contrast to the monometallic catalysts, the relative intensity of *ν*_s_(NO_2_) band (1347 cm^–1^) is significantly weaker than that of *ν*_as_(NO_2_) (1512 cm^–1^) on 0.4 wt.% RuNi (SAA), which is attributed to the cleavage of N–O bond in −NO_2_ to generate nitroso intermediate according to previous studies^49,50^. A similar phenomenon was also observed in 2 wt.% RuNi sample. The results above confirm that the synergy of bimetallic sites in RuNi SAA promotes the polarization and dissociation of nitro group, which accords well with the optimal adsorption configuration obtained by DFT calculations.

In situ XAFS spectroscopy was performed to investigate the dynamic evolution in electronic structure and coordination state of RuNi SAA during both 4-NS adsorption and catalytic reaction process. As shown in the Ru K-edge XANES spectrum (Fig. 5c), the absorption edge shifts remarkably to higher energy after adsorption of 4-NS on 0.4 wt.% RuNi SAA, accompanied with an enhanced white line intensity, indicating the Ru species undergoes partial oxidation due to the electron transfer from Ru to 4-NS. Furthermore, the phase correction EXAFS Fourier–transform spectra (Fig. 5d) of 4-NS adsorbed RuNi SAA show distinctly increased signals at ~1.7 Å and ~3.2 Å, which are ascribed to the formation of Ru–O coordination. After the introduction of H_2_ for 30 min, both the XANES and EXAFS spectra of 0.4 wt.% RuNi SAA recover to their original state. These phenomena indicate that the nitro-group adsorbs on Ru site based on electronic interaction via Ru–O bond. As for the Ni-K edge in situ XAFS spectroscopy (Supplementary Fig. 38), no obvious change is observed, which is possibly due to the dilutive effect of Ni atoms in the bulk phase on the surface Ni species. The projected density of states (PDOS) of RuNi SAA (Fig. 5e) show that the unoccupied states of nitro group over Femi level widen in adsorption state and shift below the Femi level, which further manifests electron transfer from *d*-states of RuNi SAA to the unoccupied states in −NO_2_. Charge density difference (Supplementary Fig. 39) and Bader charge analysis (Supplementary Fig. 40) display that the integral electrons density of 4-NS is accumulated after its adsorption on RuNi SAA(111) surface. Moreover, compared with the vinyl group in 4-NS, the charge density of −NO_2_ enhances more significantly by virtue of the electron transfer from Ru–Ni interface, indicating in the specific activation of the N–O bond in nitro group.

**Fig. 6:**
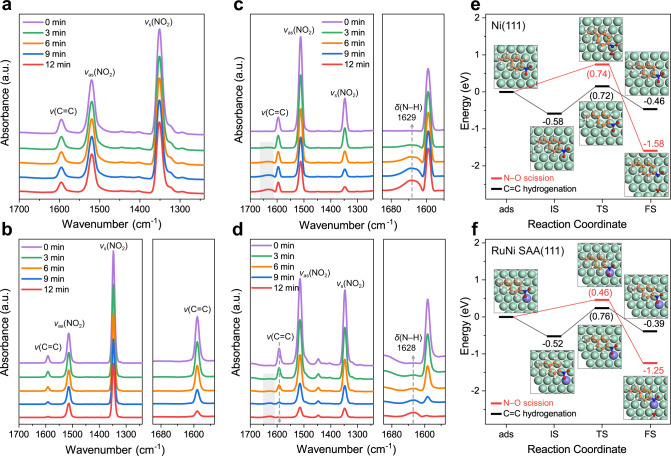
Investigations on reaction paths of 4-NS hydrogenation. In situ FT-IR spectra of 4-NS hydrogenation in the presence of **a** monometallic Ni, **b** monometallic Ru, **c** 0.4 wt.% RuNi SAA, and **d** 2 wt.% RuNi, recorded within 1700–1240 cm^–1^ by flowing H_2_ as a reaction gas after 0, 3, 6, 9, and 12 min, respectively. ‘a. u.’ denotes arbitrary units. Potential energy profiles and corresponding optimized structures for C=C hydrogenation and N–O scission in 4-NS over **e** Ni(111), and **f** RuNi SAA(111) surface. Ru, violet; Ni, green; C, orange; O, red; N, blue; H, white. ‘ads’, ‘IS’, ‘TS’ and ‘FS’ represent the adsorption state, initial state, transition state, and final state, respectively.

In situ FT-IR was performed to monitor the catalytic reaction process of 4-NS hydrogenation on catalyst samples (Fig. 6). For pristine Ni catalyst (Fig. 6a), only a slight decrease in band density of nitro groups (1520 and 1351 cm^–1^) and C=C (1595 cm^–1^) was observed along with flowing H_2_ for 12 min, indicating a rather weak activation for both −NO_2_ and C=C. In the presence of monometallic Ru (Fig. 6b), the *ν*(C=C) band (at 1591 cm^–1^) shrinks rapidly within 0−12 min whist the bands of *ν*_s_(NO_2_) and *ν*_as_(NO_2_) decline very slowly. This manifests the preferential activation hydrogenation of vinyl-group, resulting in the formation of 4-nitroethylbenzene (4-NE). In the case of 0.4 wt.% RuNi (SAA) (Fig. 6c), with the inflow of hydrogen, the band of *ν*_s_(NO_2_) (1347 cm^–1^) disappears rapidly accompanied with a gradual recession of *ν*(N=O); whilst a new band centered at 1629 cm^–1^ ascribed to N–H bending vibration is observed. Moreover, the band of *ν*(C=C) (1596 cm^–1^) merely displays a rather slight decline after 12 min. This dynamic variation verifies the polarization and hydrogenation of −NO_2_ to −NH_2_ over 0.4 wt.% RuNi (SAA), in accordance with the unique chemoselectivity toward 4-aminostyrene. For 2 wt.% RuNi sample (Fig. 6d), in addition to the appearance of a new band attributed to *δ*(N–H) at 1628 cm^–1^, the band of *ν*(C=C) decreases and fades away preferentially compared with the bands of nitro group (1514 and 1348 cm^–1^). This indicates both C=C and −NO_2_ are effectively activated on account of the existence of Ru−Ru and Ru−Ni interface sites, respectively, corresponding to the formation of 4-NE and 4-AE over 2 wt.% RuNi catalyst.

The potential conversion paths of 4-NS over Ni(111) and RuNi SAA(111), including the hydrogenation of C=C group and cleavage of N−O bond were investigated by DFT calculations, so as to further reveal the decisive role of Ru−Ni interfacial sites for the target production of 4-AS. For Ni(111) surface (Fig. 6e), the energy barriers of N−O scission and the first hydrogenation step in vinyl group are 0.74 and 0.72 eV, respectively, demonstrating that the chemoselective hydrogenation of nitro-group in 4-NS is unfavorable over monometallic Ni surface. In contrast, the energy barrier of N−O dissociation is merely 0.46 eV over RuNi SAA(111), much lower than that of C=C bond hydrogenation (0.76 eV) (Fig. 6f). This unambiguously confirms that the Ru−Ni interface sites effectively reduce the energy barrier of N−O scission in nitro group, resulting in the thermodynamically preferential reduction of nitro than C=C group on the surface of RuNi SAA, which is in accord with the consequence of experiments.

**Fig. 7:**
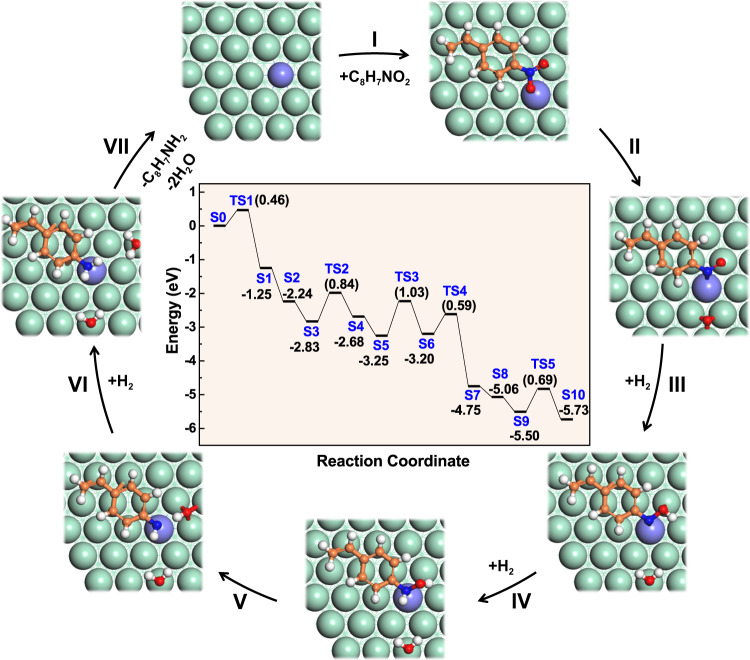
Reaction mechanism of 4-NS hydrogenation to 4-AS over RuNi SAA. A schematic illustration for the reaction mechanism of 4-NS hydrogenation to 4-AS on the surface of RuNi SAA. Ru, violet; Ni, green; C, orange; O, red; N, blue; H, white. The inset shows potential energy profiles for 4-NS hydrogenation on RuNi SAA(111) surface based on DFT calculations. ‘S0’ denotes the initial state and ‘S1–S10’ represent a series of adsorption states. ‘TS’ denotes a transition state. The numbers in parentheses stand for the energy barriers of the elementary step and the other numbers represent the adsorption energies of corresponding intermediates.

The reaction mechanism and computational energy profile (Fig. 7) of 4-NS hydrogenation on RuNi SAA were studied based on DFT calculations, and the detailed adsorption configurations for elementary steps were displayed in Supplementary Fig. 41. To optimize computational procedure, the energy barrier for the formation of water molecule was excluded from the slab model calculations^9,17^. As shown in Fig. 7, firstly, the 4-NS molecule experiences parallel absorption on RuNi SAA with the two O atoms in nitro group bond to Ru–Ni interface sites (S0; step I). Subsequently, the N–O bond connecting with Ru site undergoes breakage, accompanied with the generation of nitroso intermediate (C_8_H_7_NO*) at Ru–Ni interfacial sites and O* at Ni hollow site (S0 → S1 via TS1; energy barrier: 0.46 eV; step II). The O* radical is hydrogenated by active H atoms to form H_2_O molecule with an exotherm of 0.99 eV (S1 → S2). The hydrogenation energy barriers of C_8_H_7_NO* intermediate (Supplementary Figs. 42 and 43) indicate that the active H atoms from Ru–Ni hollow sites preferentially attack O atom rather than N atom, giving rise to C_8_H_7_NOH* (S2 → S4; energy barrier of TS2: 0.84 eV; step III). Afterwards, the N atom in C_8_H_7_NOH* is hydrogenated to produce C_8_H_7_NHOH* after overcoming a barrier of 1.03 eV (S4 → S6; step IV), which acts as the rate-determining step of the whole reaction. The N–OH bond in C_8_H_7_NHOH* further experiences scission at Ru–Ni interface sites (S6 → S7; energy barrier: 0.59 eV; step V), followed by the hydrogenation of OH* to H_2_O (S7 → S8; exotherm: 0.31 eV). Whereafter, the N atom in C_8_H_7_NH* at Ru–Ni hollow sites is further hydrogenated to yield C_8_H_7_NH_2_* (4-AS) with an energy barrier of 0.69 eV (S8 → S10; step VI). Finally, desorption of 4-AS and H_2_O molecules occur from the RuNi SAA surface and the catalyst recovers to its original state (step VII). This unique interfacial structure between Ru single atom and Ni substrate, accompanied by host-dopant synergistic effect in RuNi SAA, leads to the outstanding activity and chemoselectivity for 4-NS hydrogenation.

In summary, a RuNi SAA catalyst was prepared via galvanic replacement reaction between RuCl_3_ and Ni NPs derived from LDHs precursor. The resulting RuNi SAA displays an excellent catalytic performance (4-AS yield: >99%; TOF value: 4293 h^–1^) toward chemoselective hydrogenation of 4-NS, in comparison to monometallic Ru, Ni, and other previously reported heterogeneous catalysts. A combination characterization including AC–HAADF–STEM, in situ CO–DRIFTS and XAFS confirms that the Ru atoms are anchored onto Ni NPs at single-atom level through Ru–Ni bond, accompanied with electron transfer from Ni to Ru. In situ XAFS, FT-IR experiments and DFT calculations reveal that Ru−Ni interfacial sites serve as the intrinsic active centers to preferentially activate the N–O bond in nitro-group; and the synergistic effect between Ru and adjacent Ni sites facilitates the activation and hydrogenation of intermediates, accounting for the largely enhanced catalytic performance. This work provides atomic-level insights into the relationship between bifunctional active sites and catalytic behavior of SAA, which paves an avenue for the rational design of other bicomponent catalysts with desirable selectivity.

## Methods

### Chemicals and materials

Analytical reagents used in the experiments were bought from Sigma Aldrich: Al_2_(SO_4_)_3_·18H_2_O, sodium tartrate, CO(NH_2_)_2_, NH_4_NO_3_, Ni(NO_3_)_2_·6H_2_O, RuCl_3_, ethanol, 4-nitrostyrene (4-NS), 4-aminostyrene, 4-nitroethylbenzene, 4-aminoethylbenzene, and nitrobenzene, styrene. Purified water was adopted in all the experiments.

### Synthesis of catalysts

As a precursor, hierarchical NiAl-LDHs was synthesized by an in situ growth method^51^. Initially, urea (3.36 g), Al_2_(SO_4_)_3_·18H_2_O (9.33 g), and sodium tartrate (0.32 g) were dissolved in deionized water (140 mL). The resulting solution was transferred into a Teflon lined autoclave and heated to 170 °C for 3 h. The as-obtained precipitate was washed with distilled water and dried thoroughly, followed by a calcination at 500 °C (2 °C·min^–1^; 4 h) to obtain amorphous Al_2_O_3_. Subsequently, Al_2_O_3_ (0.2 g), Ni(NO_3_)_2_·6H_2_O (5.8 g) and NH_4_NO_3_ (9.6 g) were dispersed in purified water (200 mL), and the pH was adjusted to ~6.5 with 1 mol·L^–1^ NH_3_·H_2_O. The suspension was transferred into a flask and aged at 90 °C for 48 h to obtain NiAl-LDHs. Afterwards, NiAl-LDHs powder (0.3 g) was reduced in a H_2_/N_2_ (10/90, v/v; 35 mL·min^−1^) stream at 500 °C for 4 h (heating rate: 2 °C·min^–1^) to prepare amorphous Al_2_O_3_ supported monometallic Ni sample (Ni/Al_2_O_3_). The supported RuNi bimetallic samples were synthesized by a galvanic replacement method. Typically, the fresh Ni/Al_2_O_3_ sample (0.2 g) was dispersed in 30 mL purified water, followed by slowly adding RuCl_3_ solution (0.07 mmol·L^−1^) and stirring vigorously for 60 min under the protection of a N_2_ atmosphere. The obtained precipitation was centrifugated, washed with purified water, and dried for 24 h in vacuum oven at 50 °C to obtain 0.1% RuNi sample. Before the catalytic evaluation, the as-synthesized samples were pre-reduced in a H_2_/N_2_ flow (10/90, v/v) at 300 °C (heating rate: 2 °C·min^–1^) for 1 h, followed by cooling to the room temperature in N_2_. As references, 0.4 wt.% and 2 wt.% Ru/Al_2_O_3_ samples, with an actual Ru content of 0.36 wt.% and 2.3 wt.% respectively, were prepared by a deposition precipitation method and reduced at 300 °C (H_2_/N_2_ flow: 10/90, v/v; heating rate: 2 °C·min^–1^) for 3 h.

### Characterizations

The X-ray diffraction (XRD) experiments were carried out on Bruker DAVINCI D8 ADVANCE diffractometer with a Cu Kα radiation source (40 kV and 40 mA). Shimadzu ICPS-7500 inductively coupled plasma−atomic emission spectrometer (ICP−AES) instrument was used to determine the actual element contents of various samples. Scanning electron microscope (SEM) images were displayed by using a Zeiss Supra 55 electron microscope. N_2_ adsorption–desorption experiments were performed on a Micromeritics ASAP 2020 device, and multipoint Brunauer–Emmett–Teller (BET) method was used to calculate the specific surface area. Transmission electron microscopy (TEM) characterizations were performed on a JEOL JEM-2010 high-resolution transmission electron microscope. FEI Titan Cube Themis G2 300 and JEOL JEM-ARM200F instruments with a spherical aberration corrector and energy-dispersive X-ray spectroscopy (EDS) system were adopted to perform aberration-corrected high angle annular dark-field scanning transmission electron microscopy (AC−HAADF−STEM) and EDS mapping measurements. In situ X-ray absorption fine structure spectroscopy (XAFS) at Ru K-edge and Ni K-edge were measured at the beamline 1W1B and 1W2B of the Beijing Synchrotron Radiation Facility (BSRF), Institute of High Energy Physics (IHEP), Chinese Academy of Sciences (CAS). CO pulse chemisorption and hydrogen temperature-programmed desorption (H_2_-TPD) experiments were carried out on a Micromeritics Autochem II 2920 instrument using a thermal conductivity detector (TCD). In situ DRIFTS and FT-IR experiments were conducted on a Bruker TENSOR II infrared spectrometer equipped with a modified in situ reaction cell and MCT highly-sensitive detector. The detailed characterization methods are described in the Supplementary Information.

### Catalytic test

Firstly, substrate (4-NS, 1 mmol), solvent (ethanol, 8 ml), and catalyst (0.02 g) were carefully added to a 25 mL stainless-steel autoclave. Subsequently, the reactor was purged completely with 2.0 MPa hydrogen (>99.999%) for 5 times, followed by pressurized and sealed with H_2_ to 1.0 MPa. The reaction was carried out at 60 °C with a constant stirring speed of 700 rpm. After the reaction is over, the resulting products were identified by GC−MS, and quantitatively analyzed using a Shimadzu GC-2014C gas chromatograph system outfitted a GSBP-INOWAX capillary column (30 m × 0.25 mm × 0.25 mm) and an FID detector. The conversion of 4-nitrostyrene and the selectivity of products were determined as follows:

1 $${{{{{\rm{Conversion}}}}}}( \% )=\left(1-\frac{{{{{{\rm{Mole}}}}}}\,{{{{{\rm{number}}}}}}\,{{{{{\rm{of}}}}}}\,4-{{{{{\rm{NS}}}}}}\,{{{{{\rm{after}}}}}}\,{{{{{\rm{reaction}}}}}}}{{{{{{\rm{Initial}}}}}}\,{{{{{\rm{mole}}}}}}\,{{{{{\rm{number}}}}}}\,{{{{{\rm{of}}}}}}\,4-{{{{{\rm{NS}}}}}}\,{{{{{\rm{fed}}}}}}}\right)\times 100 \%$$

2 $${{{{{\rm{Selectivity}}}}}}( \% )=\frac{{{{{{\rm{Mole}}}}}}\,{{{{{\rm{number}}}}}}\,{{{{{\rm{of}}}}}}\,{{{{{\rm{one}}}}}}\,{{{{{\rm{product}}}}}}}{{{{{{\rm{Total}}}}}}\,{{{{{\rm{mole}}}}}}\,{{{{{\rm{number}}}}}}\,{{{{{\rm{of}}}}}}\,4-{{{{{\rm{NS}}}}}}\,{{{{{\rm{converted}}}}}}}\times 100 \%$$

The turnover frequency (TOF) values were calculated as the molar amount of 4-NS converted per mole of metal sites per hour (mol_4-NS_ mol^−1^ h^−1^), under a low 4-NS conversion (~15%) based on the number of Ru sites, the Ru−Ni interfacial sites and total surface metal atoms. For the reusability tests, the catalyst was collected by centrifugation after reaction, and washed with ethanol for three times, followed by re-adding into the autoclave for the next catalytic cycle.

### DFT calculations

All the density functional theory (DFT) calculations were performed using the Vienna ab initio simulation package (VASP 5.4.1). The PBE functional with generalized gradient approximation (GGA) was used to describe the electron exchange and correlation term. The projector augmented wave (PAW) method was employed to describe the interaction between atomic cores and electrons. The effect of van der Waals interactions between the substrates and the interface were described by Grimme’s DFT-D3 method. The energy barriers were calculated by the climbing image nudged elastic band (CI-NEB) and Dimer methods. Vibrational frequency analysis was performed, which verified only one imaginary frequency was present in each transition state (Supplementary Figs. 44–51). More detailed computational methods are described in the Supplementary Information.

## Supplementary information

Supplementary Information

## Data Availability

The primary data that support the plots within this paper are provided in the Source Data file. Additional data related to this study are available from the corresponding authors upon reasonable request. Source data are provided with this paper.

## Source data

Source data
