# Supplementary material for: Highly-efficient RuNi single-atom alloy catalysts toward chemoselective hydrogenation of nitroarenes
Source: Nat Commun. 2022 Jun 8;13:3188. doi: 10.1038/s41467-022-30536-9 (PMC9178046; doi:10.1038/s41467-022-30536-9)
Supplement: Supplementary file 1 — Supplementary Information [file 41467_2022_30536_MOESM1_ESM.pdf]

Supplementary Information

Highly-efficient RuNi single-atom alloy catalysts toward chemoselective hydrogenation of nitroarenes

Wei Liu[1], Haisong Feng[1], Yusen Yang[1]*, Yiming Niu[2], Lei Wang[1], Pan Yin[1], Song Hong[1], Bingsen Zhang[2], Xin Zhang[1]*, Min Wei[1]*

[1] State Key Laboratory of Chemical Resource Engineering, Beijing Advanced Innovation Center for Soft Matter Science and Engineering, Beijing University of Chemical Technology, Beijing 100029, P. R. China

[2] Shenyang National Laboratory for Materials Science, Institute of Metal Research, Chinese Academy of Sciences, Shenyang 110016, P. R. China

Author Information

* Corresponding authors. Tel: +86-10-64412131; Fax: +86-10-64425385.

E-mail addresses: yangyusen@mail.buct.edu.cn (Y. Yang); zhangxin@mail.buct.edu.cn (X. Zhang); weimin@mail.buct.edu.cn (M. Wei).

Supplementary Methods

In situ XAFS measurements

In situ X-ray absorption fine structure spectroscopy (XAFS) at the Ru K-edge (fluorescence mode) and Ni K-edge (transmission mode) was carried out at the beamline 1W1B and 1W2B of the Beijing Synchrotron Radiation Facility (BSRF), Institute of High Energy Physics (IHEP), Chinese Academy of Sciences (CAS). Typically, the powdered sample was pressed into a self-supporting wafer and carefully placed into a reaction microdevice equipped with polyimide windows. Afterwards, the sample was pre-reduced in a H_2/He flow (1/19, v/v; 30 mL·min^{-1}) at 300 °C for 1 h, and then cooled to room temperature in a high-purity He stream to collect in situ XAFS spectra. In the 4-NS adsorption experiment, based on the above pretreatment, a certain amount of 4-NS was carefully evaporated into the in situ cell in He flow (30 mL·min^{-1}) at 60 °C, followed by flowing pure He to remove physically adsorbed 4-NS; afterwards, XAFS spectra were collected. Finally, the gas flow was switched to a H_2/He (1/19, v/v) atmosphere to trigger the surface reaction and then XAFS spectra were collected. All the XAFS data were processed using Athena software package[1]. Wavelet transformation for the Ru K-edge XAFS signals was employed based on Morlet wavelets[2].

In situ DRIFTS and FT-IR measurements

In situ DRIFTS and FT-IR experiments were conducted on a Bruker TENSOR II equipped with an MCT narrow-band detector and a modified in situ reaction cell. The detailed pre-treatment and test conditions are given as follows. As for CO–DRIFTS measurements, about 50 mg of sample was carefully put into the support sink of diffuse reflectance cell firstly. Subsequently, the sample was pre-reduced in a H_2/He flow (1/19, v/v; 30 mL·min^{-1}) at 300 °C (heating rate: 5

°C·min^{-1}) for 1 h, followed by cooling to the room temperature in a high purity He stream, and then the background signal was collected. Afterwards, the CO/He (1/19, v/v; 30 mL·min^{-1}) was purged into the cell, and DRIFTS spectra were collected until the adsorption spectrums kept unchanged. Finally, the gas flow was switched to a pure He stream to collect CO chemisorption spectra. In situ FT-IR measurements of 4-NS adsorption and surface reaction were performed using a transmission reactor. The sample (20 mg) was pressed into self-supporting wafer with a diameter of 13 mm, followed by a pretreatment under the same conditions. After the sample was cooled down to 60 °C in He stream, 4-NS was introduced into the reactor for 30 min. Subsequently, He was purged to remove the physically adsorbed molecule followed by collection of IR signals. Finally, the spectra for hydrogenation process were collected per 60 s after the introduction of H_2 (flow rate: 30 mL·min^{-1}).

Computational details

We performed first-principle calculation within the DFT methodology by using the Vienna ab initio simulation package (VASP 5.4)[3,4]. The exchange and correlation energy were calculated with the generalized gradient approximation (GGA) of the density functional theory. The core electrons are described with the projector augmented wave (PAW) method[5,6]. Lattice parameters of bulk Ni were optimized by the PBE-D3[7,8], PBEsol[9], PBE[10] and PW91[11] functions. The results (Supplementary Table 6) show that the lattice parameters obtained with the PBE-D3 function accord well with the experimental values. Based on previous work[12], PBE-D3 function can be used to study the catalytic reaction process satisfactorily, so we choose PBE-D3 function for the subsequent computational studies. A 2 × 2 × 1 Monkhorst-Pack *k* point mesh and a cutoff energy of 400 eV for the plane-wave basis were employed for the geometry optimizations. The

convergence criterion for the total energy self-consistent iterations was 10^{-4} eV, and the geometry optimization stopped when the total force on the system was less than 0.05 eV/Å. The energy barriers were determined by the climbing image nudged elastic band (CI-NEB) and Dimer method[13,14].

According to the HRTEM characterization, the Ni(111) surface was modeled with a three-layer slab in a $p(6 \times 6)$ surface unit cell consisting of 108 atoms; the bottom one slab was fixed while the top two slabs were relaxed. A vacuum space of 15 Å ensures no interaction between the periodically repeated slabs or adsorbates in the direction normal to the slab. As Ni is not a non-magnetic metal, we performed spin polarization calculation for the Ni(111) surface. The RuNi(111) surface was built by substituting one Ni atom on the topmost layer with one Ru atom.

The activation energy (E_a) and adsorption energy (E_{ads}) are calculated by the following equations:

$$E_a = E_{TS} - E_{IS} \quad (1)$$

$$E_{ads} = E_{A/M} - E_A - E_M \quad (2)$$

where E_{TS}, E_{IS}, $E_{A/M}$, E_A and E_M represent the energies of the TS (transition state), IS (initial state), adsorbed system, substrate, and adsorbate species, respectively.

The Bader Charge analysis was performed via Henkelman programme based on near-grid algorithm with refine-edge method[15–17]. The charge density differential analysis was performed by using visualization software VESTA[18], with the isosurface level set as 0.005 e/Å^3.

Supplementary Figures

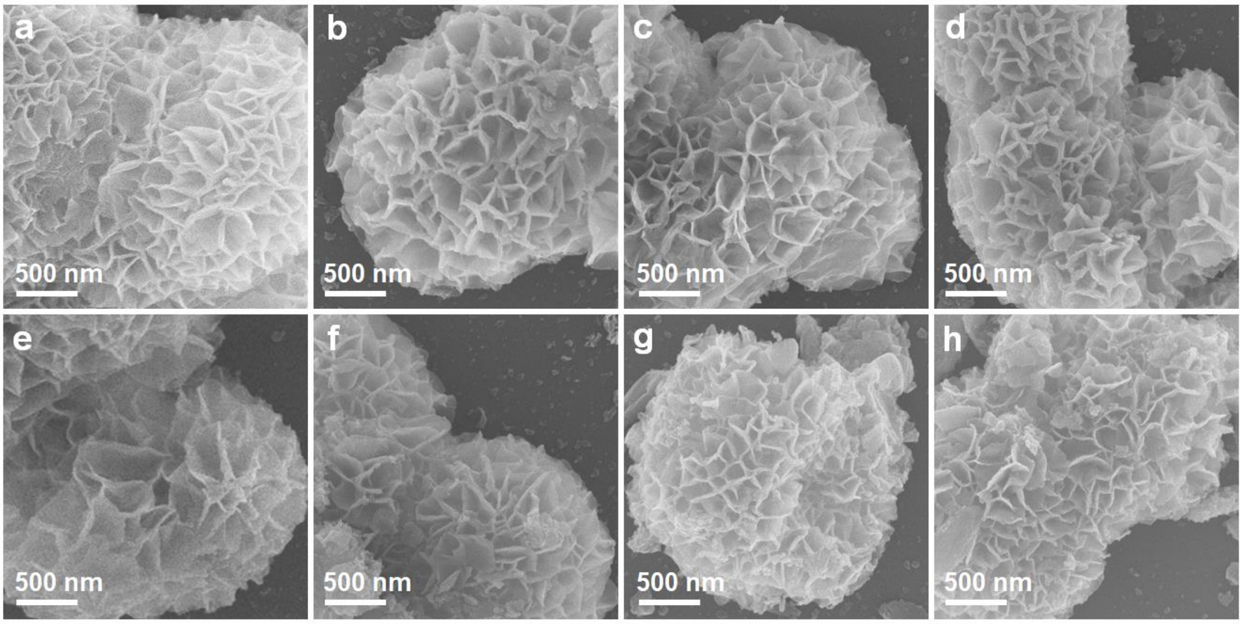

Supplementary Figure 1 | Morphology characterizations of RuNi bimetallic samples. SEM images of **a–h** Ni/Al_2O_3, 0.1 wt.%, 0.2 wt.%, 0.4 wt.%, 0.6 wt.%, 0.8 wt.%, 1 wt.% and 2 wt.% RuNi samples, respectively.

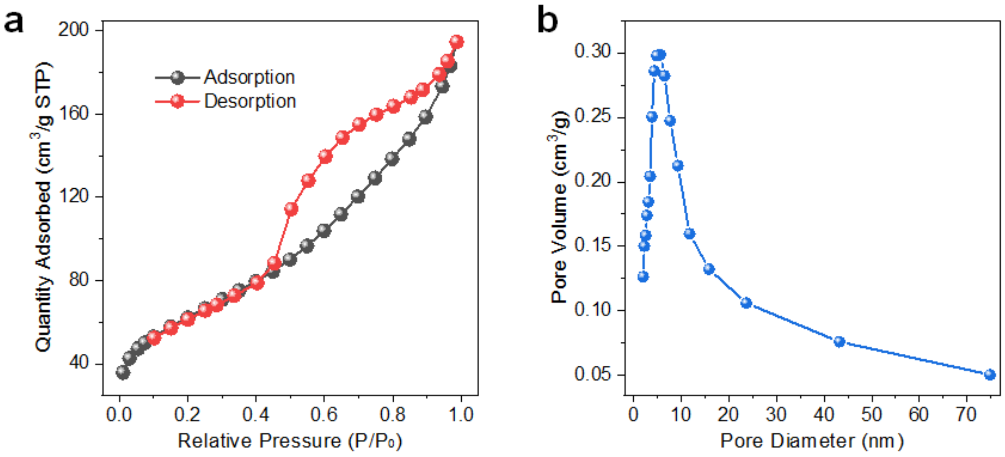

Supplementary Figure 2 | N_2 adsorption–desorption experiment of Ni/Al_2O_3. a Nitrogen adsorption-desorption isotherm and **b** the pore size distribution profile of Ni/Al_2O_3 sample.

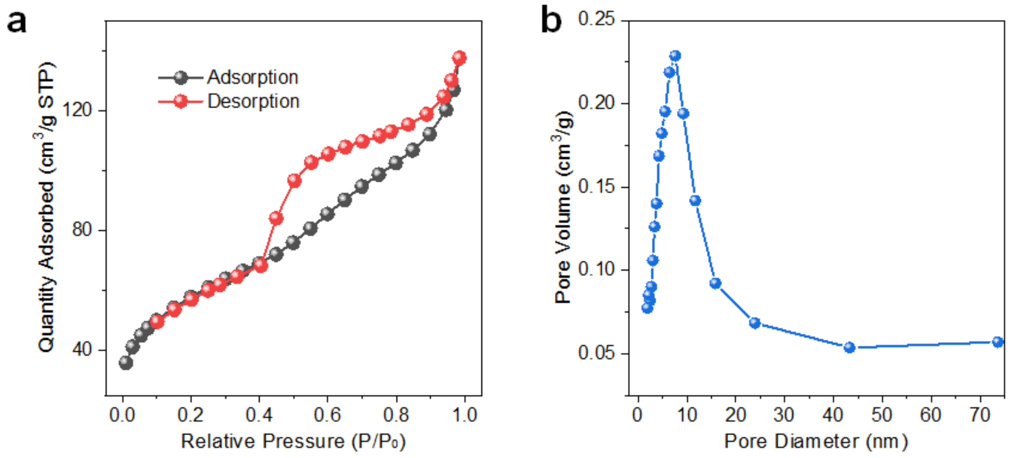

Supplementary Figure 3 | N_2 adsorption–desorption experiment of 0.1% RuNi. a Nitrogen adsorption-desorption isotherm and **b** the pore size distribution profile of 0.1 wt.% RuNi sample.

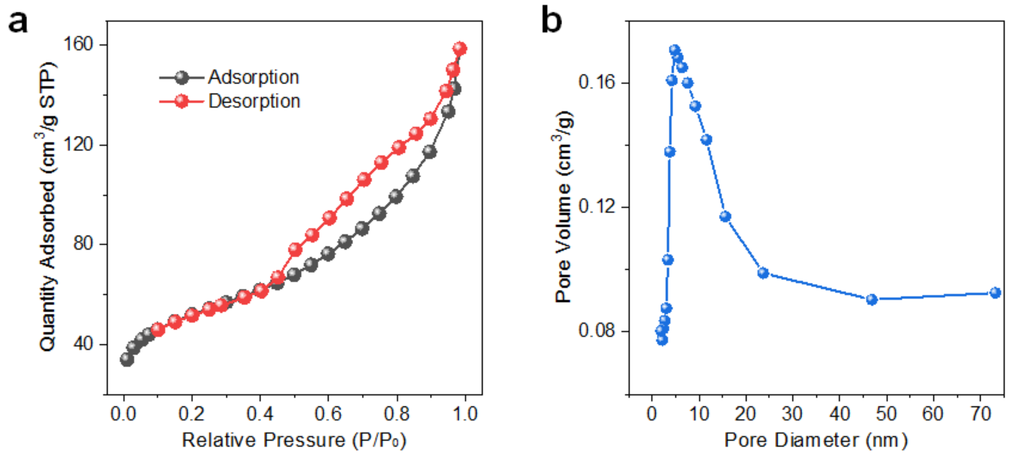

Supplementary Figure 4 | N_2 adsorption–desorption experiment of 0.2% RuNi. a Nitrogen adsorption-desorption isotherm and **b** the pore size distribution profile of 0.2 wt.% RuNi sample.

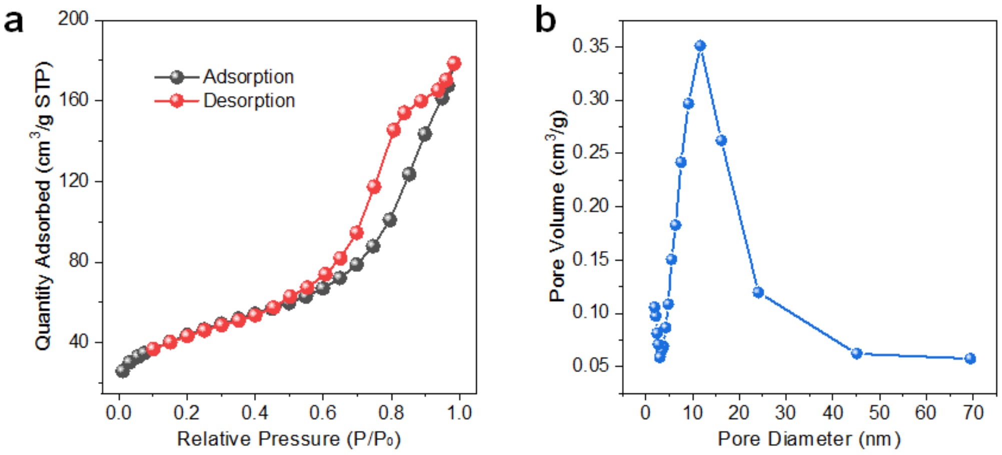

Supplementary Figure 5 | N_2 adsorption–desorption experiment of 0.4% RuNi. a Nitrogen adsorption-desorption isotherm and **b** the pore size distribution profile of 0.4 wt.% RuNi sample.

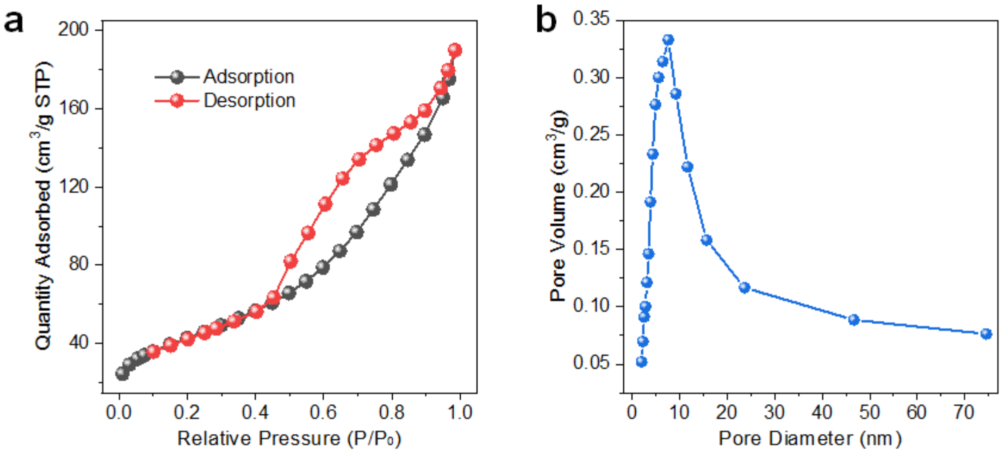

Supplementary Figure 6 | N_2 adsorption–desorption experiment of 0.6% RuNi. a Nitrogen adsorption-desorption isotherm and **b** the pore size distribution profile of 0.6 wt.% RuNi sample.

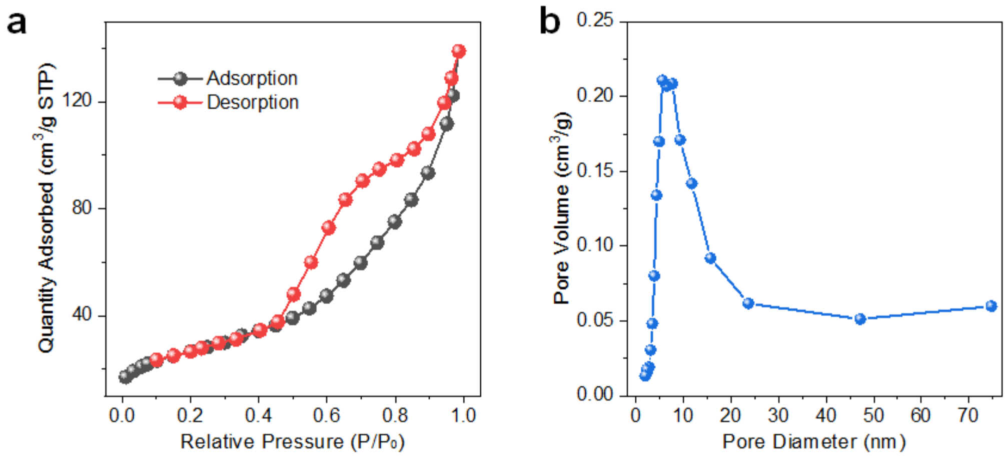

Supplementary Figure 7 | N_2 adsorption–desorption experiment of 0.8% RuNi. a Nitrogen adsorption-desorption isotherm and **b** the pore size distribution profile of 0.8 wt.% RuNi sample.

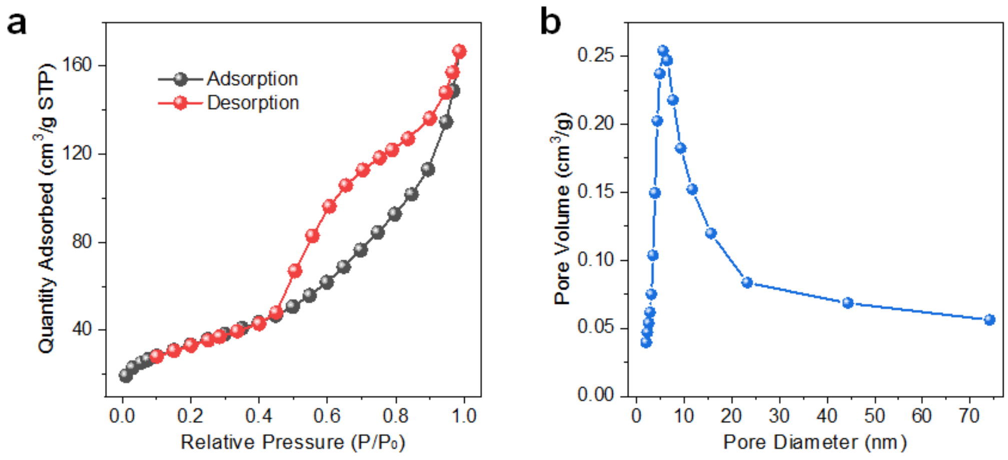

Supplementary Figure 8 | N_2 adsorption–desorption experiment of 1% RuNi. a Nitrogen adsorption-desorption isotherm and **b** the pore size distribution profile of 1 wt.% RuNi sample.

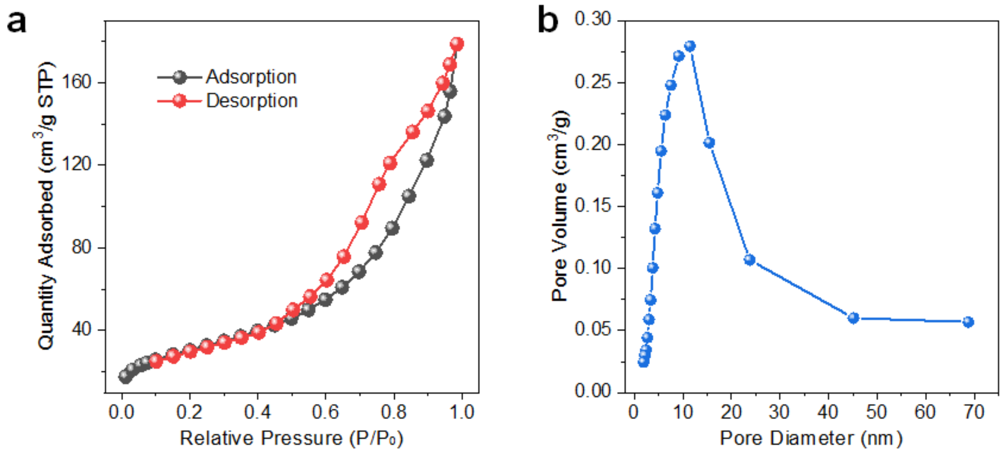

Supplementary Figure 9 | N_2 adsorption–desorption experiment of 2% RuNi. a Nitrogen adsorption-desorption isotherm and **b** the pore size distribution profile of 2 wt.% RuNi sample.

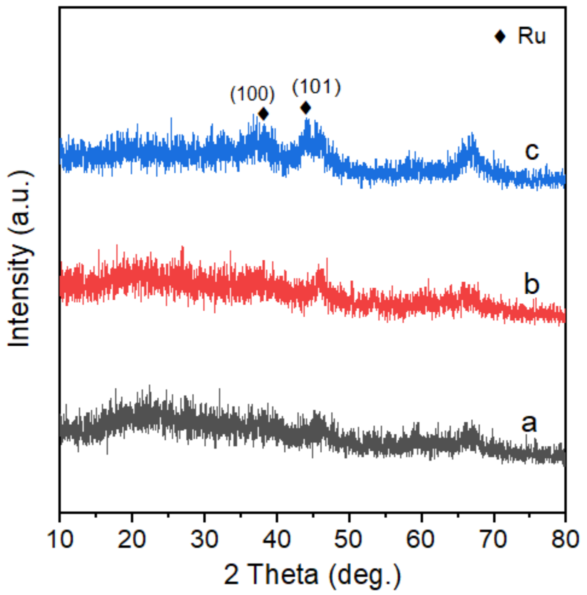

Supplementary Figure 10 | Structure characterization of various samples. XRD patterns of **a** pristine Al_2O_3, **b** 0.4 wt.% Ru/Al_2O_3 and **c** 2 wt.% Ru/Al_2O_3 samples. 'a. u.' denotes arbitrary units.

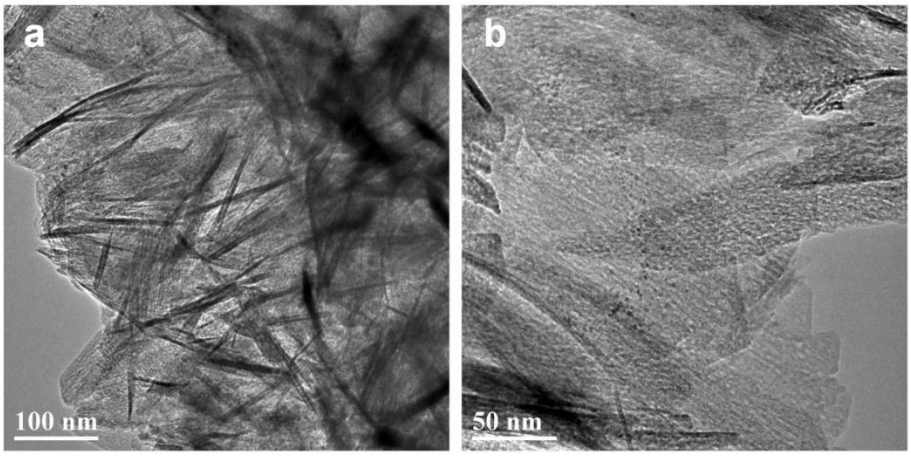

Supplementary Figure 11 | Structure characterization of 2% Ru/Al_2O_3. a TEM and **b** HRTEM images of 2 wt.% Ru/Al_2O_3 sample.

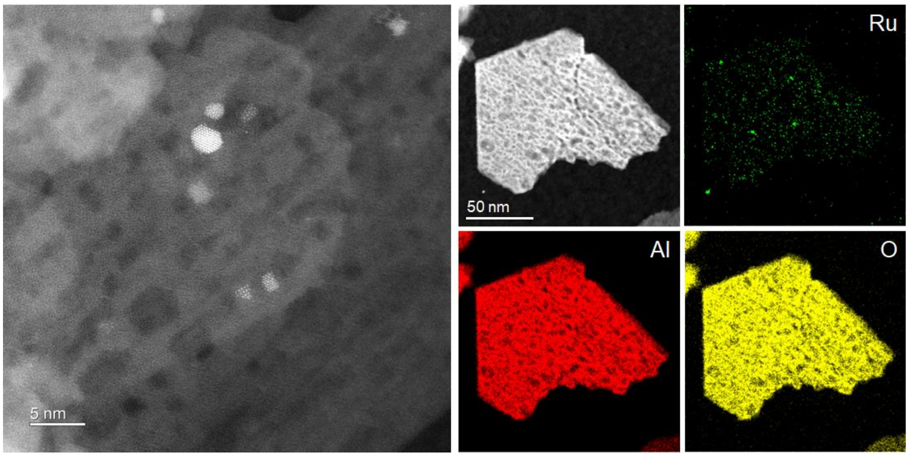

Supplementary Figure 12 | Electron micrology study on 2% Ru/Al_2O_3. HAADF−STEM image and corresponding elemental EDS mapping of 2 wt.% Ru/Al_2O_3 sample.

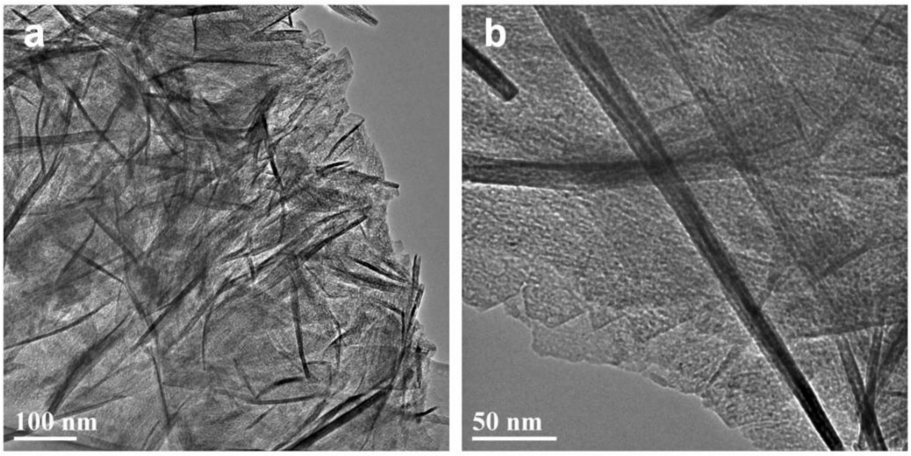

Supplementary Figure 13 | Structure characterization of 0.4% Ru/Al_2O_3. **a** TEM and **b** HRTEM images of 0.4 wt.% Ru/Al_2O_3 sample.

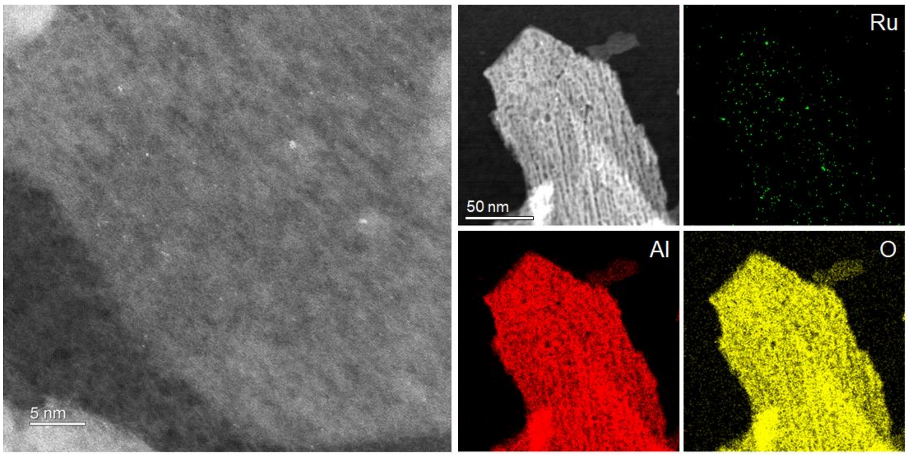

Supplementary Figure 14 | Electron micrology study on 0.4% Ru/Al_2O_3. HAADF−STEM image and corresponding elemental EDS mapping of 0.4 wt.% Ru/Al_2O_3 sample.

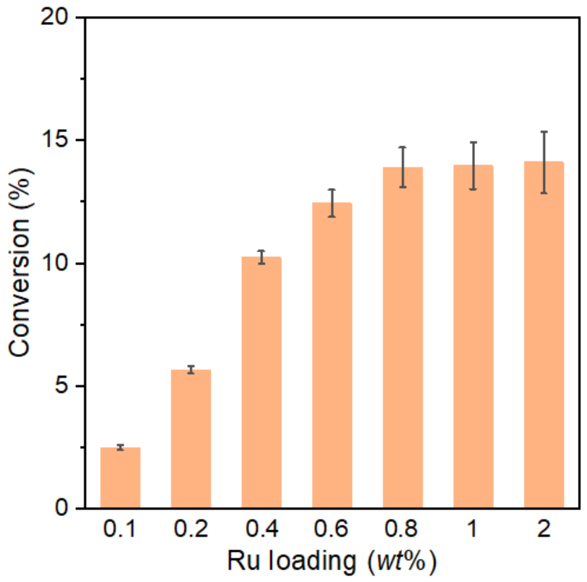

Supplementary Figure 15 | Catalytic activity of RuNi bimetallic samples. Conversion of 4-nitrostyrene over RuNi catalysts with various Ru loading (0.1–2 wt.%) after 2.5 min. Reaction conditions: 1 mmol of reactant; 8 ml of solvent (ethanol); 0.02 g of catalyst; 1 Mpa of H_2. Error bars were defined as the standard deviation by three replicate experiments.

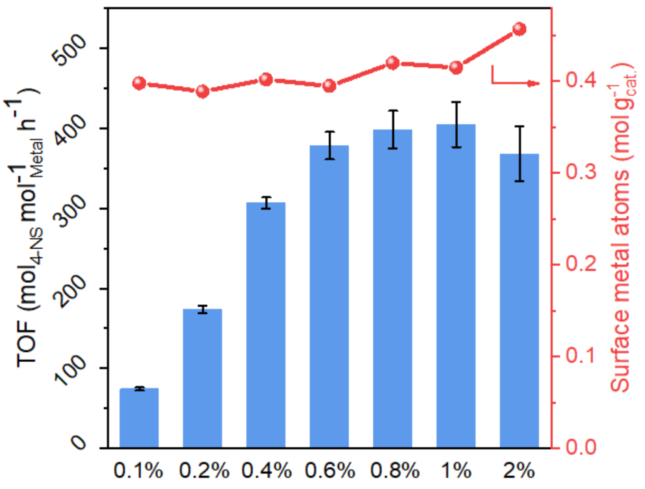

Supplementary Figure 16 | The relationship between structure and catalytic activity. Total surface metal sites and corresponding turnover frequency (TOF_{metal}) of RuNi catalysts with various Ru loading (0.1–2 wt.%). Error bars were defined as the standard deviation by three replicate experiments.

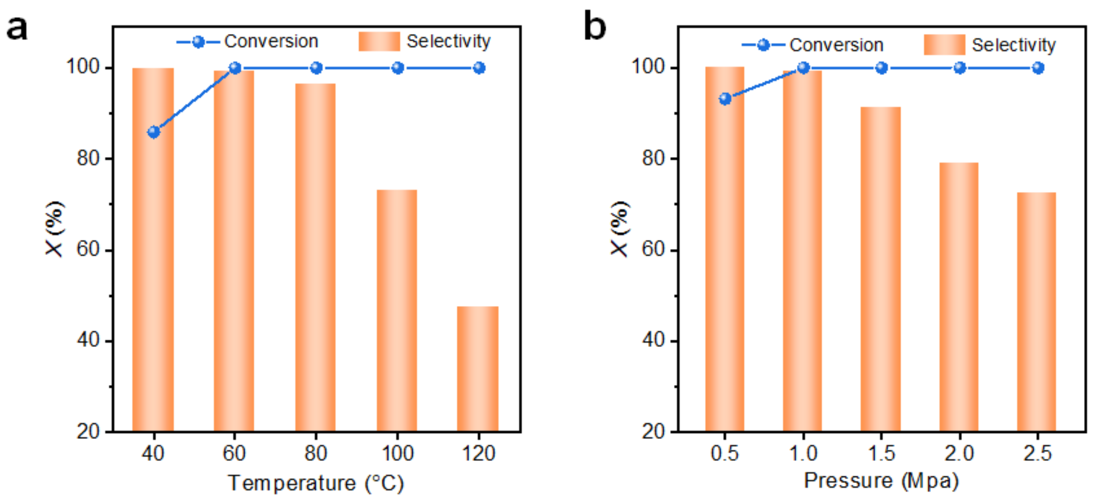

Supplementary Figure 17 | Optimizations of reaction conditions. Correlation between catalytic performance of 0.4 wt.% RuNi (SAA) sample and reaction conditions: 4-nitrostyrene conversion and 4-aminostyrene selectivity as function of **a** reaction temperature and **b** H_2 pressure, respectively.

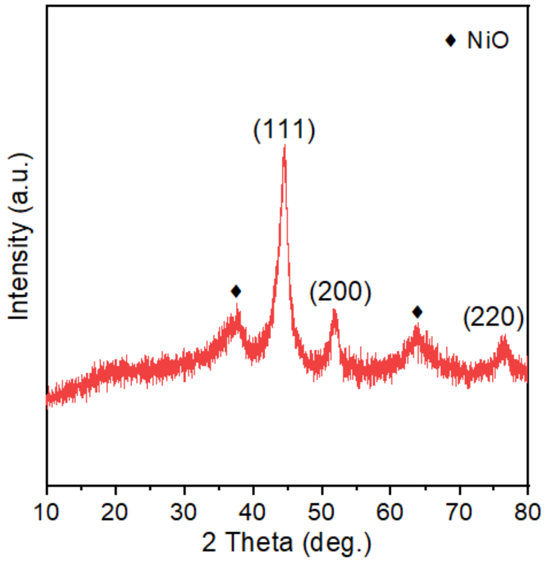

Supplementary Figure 18 | XRD pattern of the used 0.4% RuNi catalyst after 5 cycle times. Two diffraction peaks at 2θ 37.2° and 62.8° are ascribed to the (111) and (220) reflections of a NiO phase (JCPDS 047–1049), due to the insufficient reduction of Ni species from LDHs precursor, which is also present in the XRD pattern of 0.4 wt.% RuNi SAA. ‘a. u.’ denotes arbitrary units.

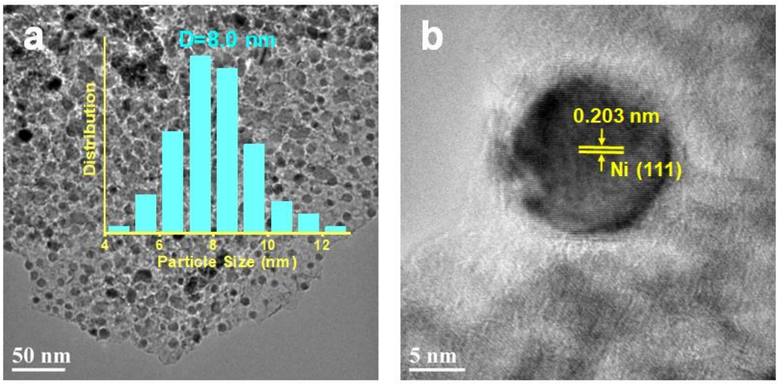

Supplementary Figure 19 | Structure characterization of the used 0.4% RuNi catalyst. a TEM image with corresponding particle size distribution and **b** HRTEM lattice fringe image of 0.4 wt.% RuNi catalyst after 5 cycle times.

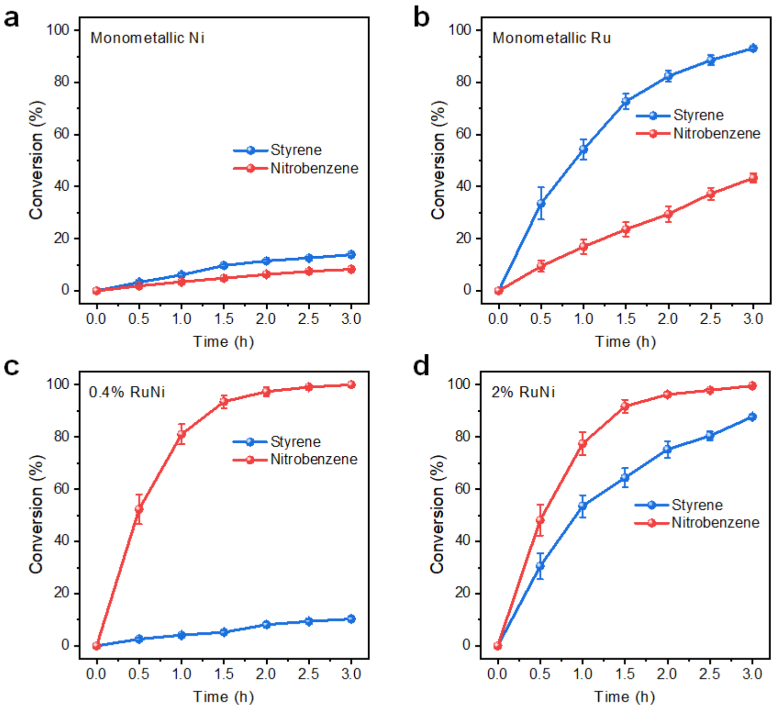

Supplementary Figure 20 | The catalytic behavior of various samples. Catalytic performance of **a** monometallic Ni, **b** monometallic Ru, **c** 0.4 wt.% and **d** 2 wt.% RuNi catalyst, toward the hydrogenation of nitrobenzene and styrene mixture (1:1), which possesses a single nitro group and a single C=C group, respectively. Reaction conditions: 1 mmol of nitrobenzene and 1 mmol of styrene; 8 ml of solvent (ethanol); 0.02 g of catalyst; 1 Mpa of H_2, 60 °C, 3 h. Error bounds were defined as the standard deviation by three replicate experiments.

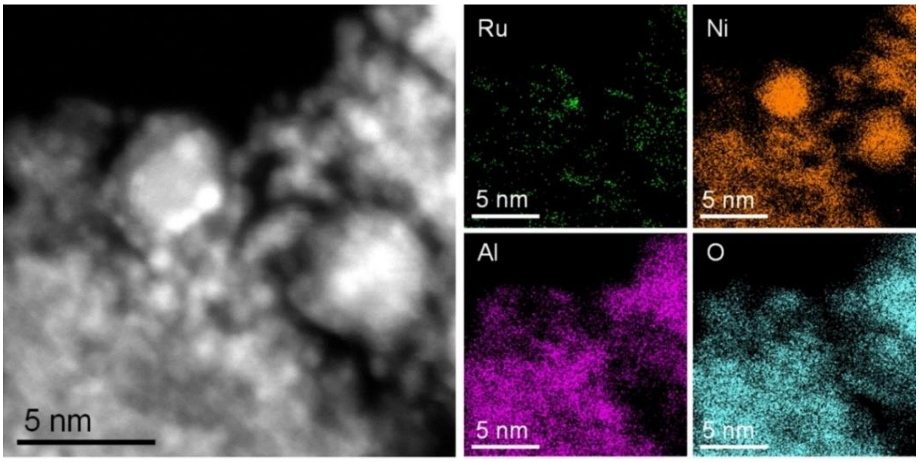

Supplementary Figure 21 | Element mapping images of 2% RuNi catalyst. HAADF−STEM image and corresponding elemental EDS mapping of 2 wt.% RuNi sample.

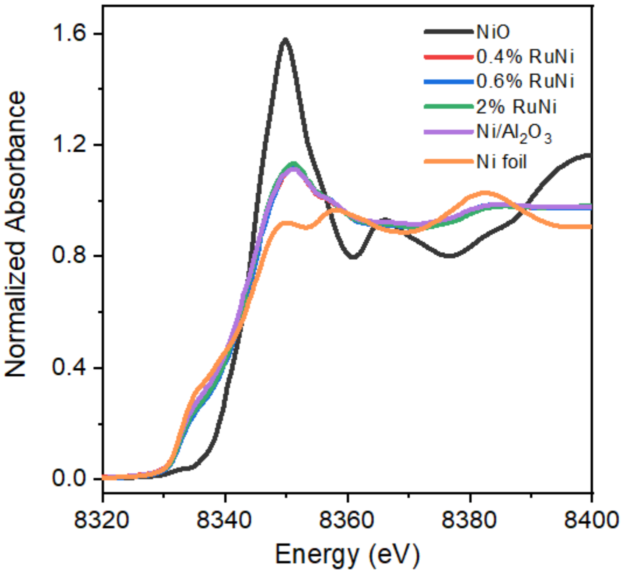

Supplementary Figure 22 | In situ XANES characterization of various samples. In situ Ni K-edge XANES spectra of Ni/Al_2O_3, 0.4 wt.%, 0.6 wt.%, 2 wt.% RuNi samples, with Ni foil, NiO as reference samples.

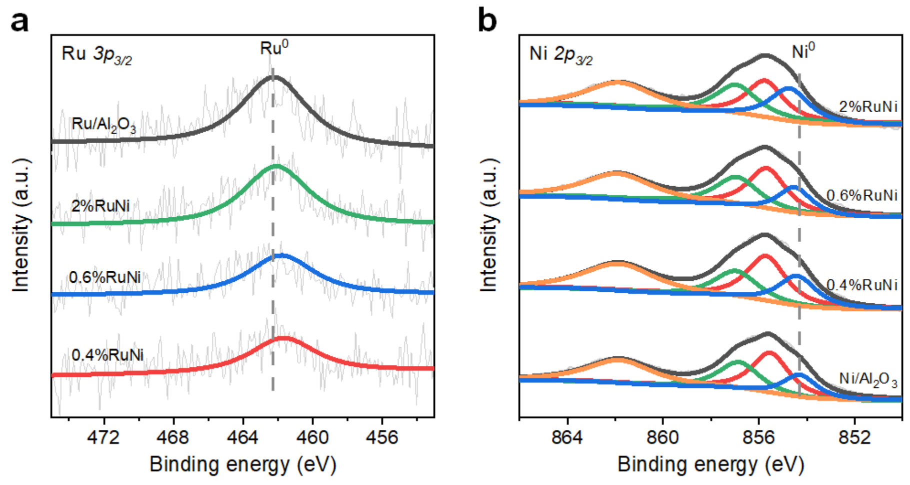

Supplementary Figure 23 | XPS spectra of various samples. a Ru *3p*$_{3/2}$ and **b** Ni *2p*$_{3/2}$ of Ru/Al_2O_3, Ni/Al_2O_3, 0.4 wt.% RuNi, 0.6 wt.% RuNi and 2 wt.% RuNi, respectively. 'a. u.' denotes arbitrary units.

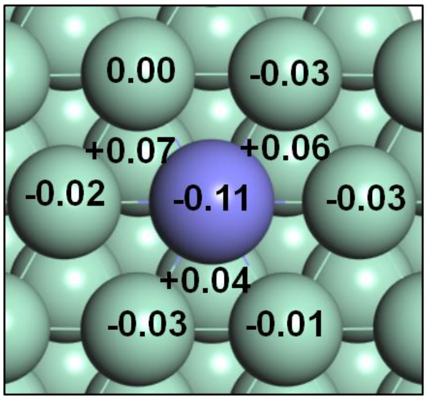

Supplementary Figure 24 | Bader charge analysis of RuNi SAA. The numbers stand for the amount of charge carried by corresponding atoms of pristine RuNi SAA(111) surface, which reveal that isolated Ru atom carries negative charges from sub-surface Ni atoms. Ru, violet; Ni, green.

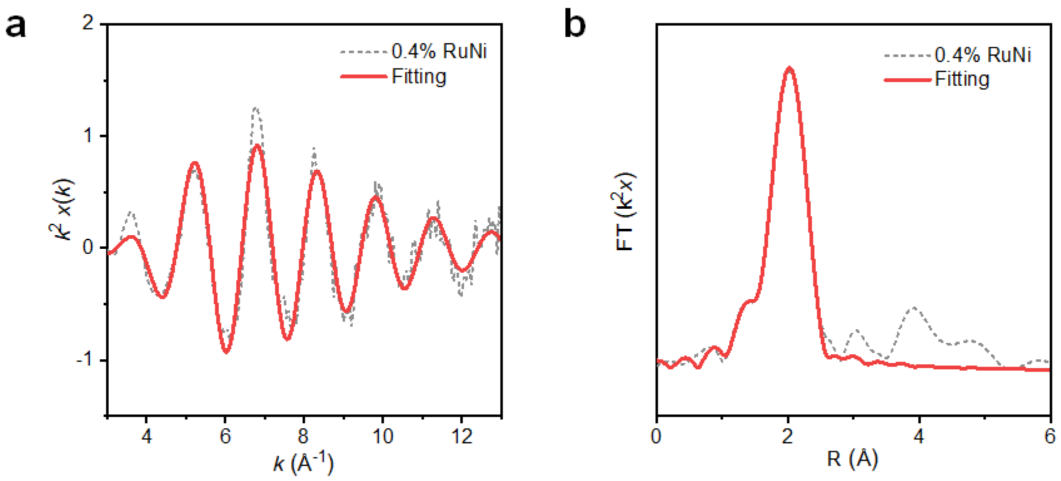

Supplementary Figure 25 | EXAFS curve-fitting for 0.4% RuNi. Fourier–transform EXAFS fitting spectra of 0.4 wt.% RuNi sample at **a** k-space and **b** R-space, respectively.

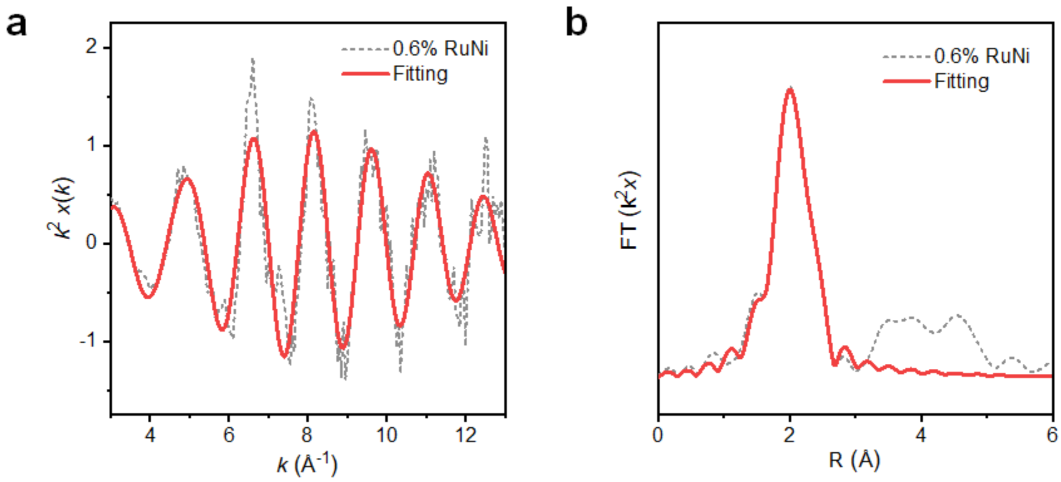

Supplementary Figure 26 | EXAFS curve-fitting for 0.6% RuNi. Fourier–transform EXAFS fitting spectra of 0.6 wt.% RuNi sample at **a** k-space and **b** R-space, respectively.

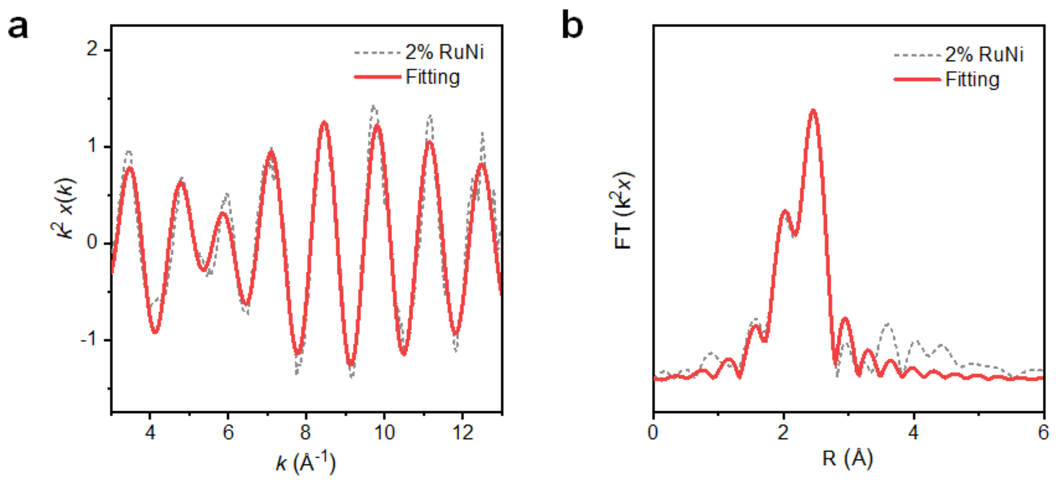

Supplementary Figure 27 | EXAFS curve-fitting for 2% RuNi. Fourier–transform EXAFS fitting spectra of 2 wt.% RuNi sample at **a** k-space and **b** R-space, respectively.

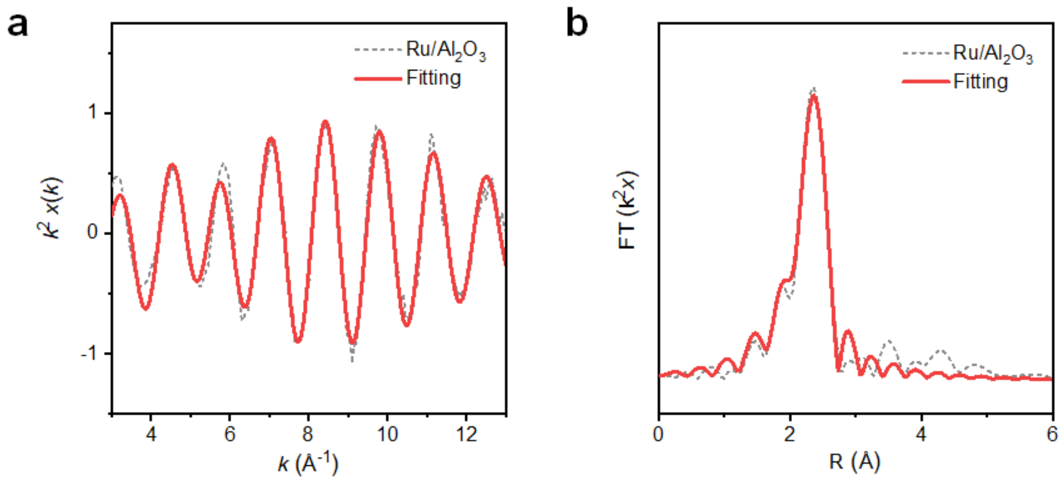

Supplementary Figure 28 | EXAFS curve-fitting for Ru/Al_2O_3. Fourier–transform EXAFS fitting spectra of 2% Ru/Al_2O_3 sample at **a** k-space and **b** R-space, respectively.

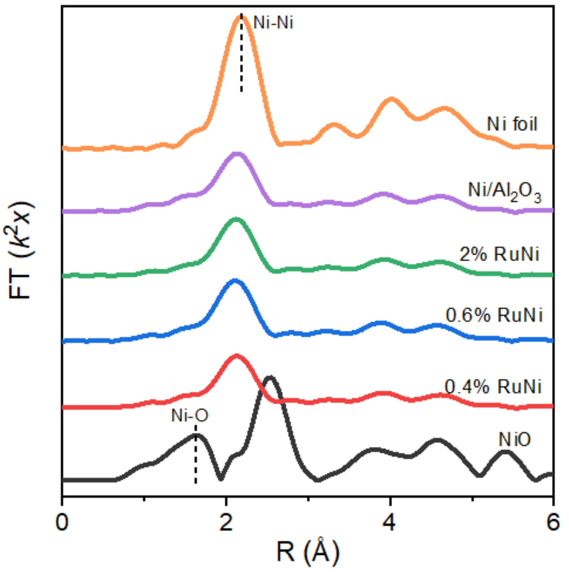

Supplementary Figure 29 | Fine-structure characterizations of various samples. In situ Ni K-edge EXAFS Fourier−transform spectra of Ni/Al_2O_3, 0.4 wt.%, 0.6 wt.%, 2 wt.% RuNi samples, and corresponding Ni foil, NiO references.

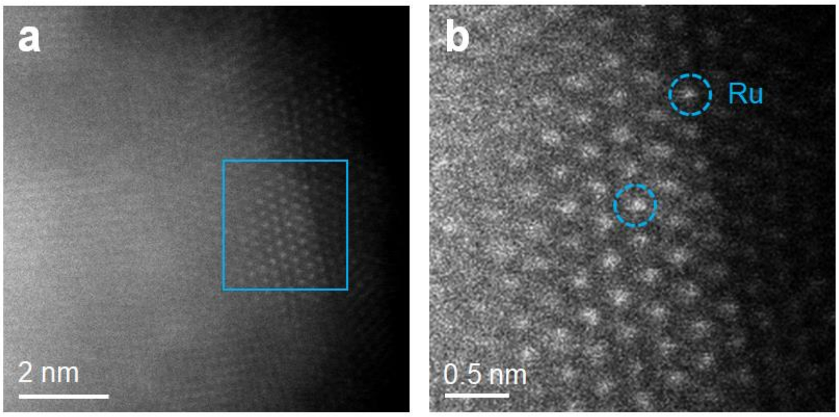

Supplementary Figure 30 | Electron micrology studies on the used RuNi SAA. a Aberration-correction HAADF−STEM image, and **b** corresponding enlarged image of the used 0.4 wt.% RuNi sample after 5 catalytic cycles.

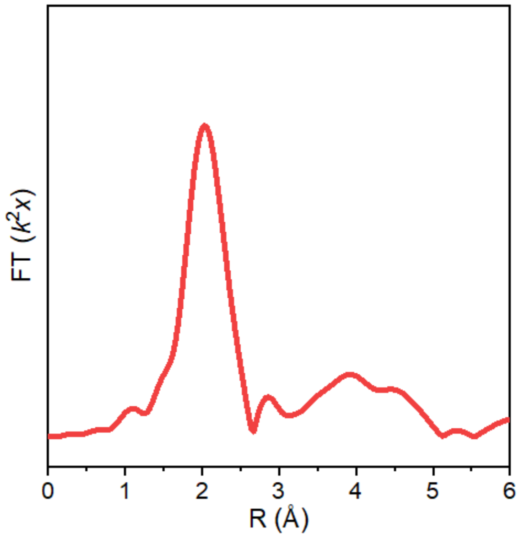

Supplementary Figure 31 | Fine-structure characterization of the used RuNi SAA. Ru K-edge EXAFS Fourier–transform spectrum of the used 0.4 wt.% RuNi sample after 5 catalytic cycles.

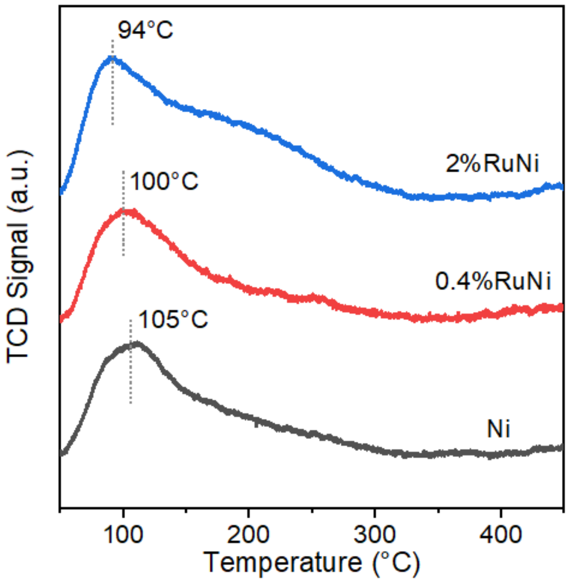

Supplementary Figure 32 | H_2-TPD experiment. H_2-TPD profiles of monometallic Ni, 0.4 wt.% RuNi (SAA), and 2 wt.% RuNi, respectively. 'a. u.' denotes arbitrary units.

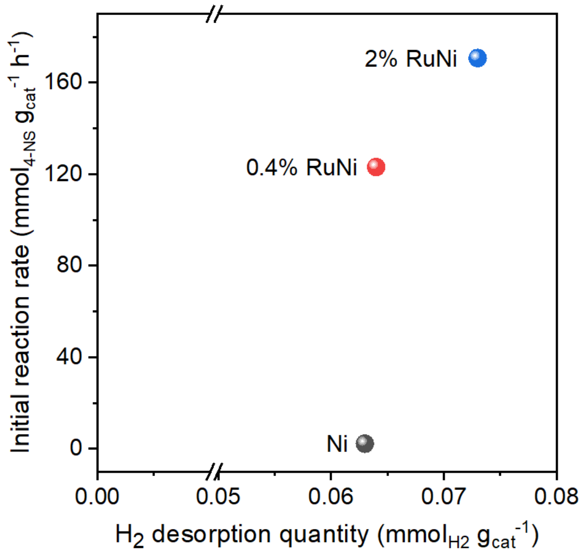

Supplementary Figure 33 | The relationship between reactivity and H_2 dissociation behavior. Initial reaction rate of 4-NS as a function of hydrogen desorption quantity over monometallic Ni, 0.4 wt.% RuNi and 2 wt.% RuNi, respectively.

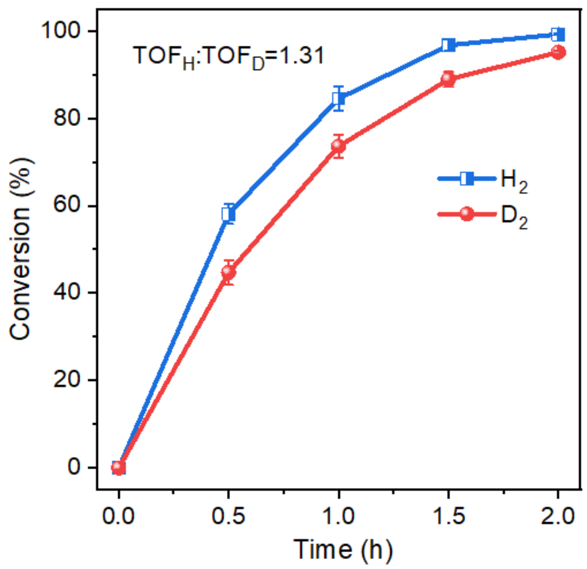

Supplementary Figure 34 | KIE experiment. Kinetic isotope effect measured for 0.4 wt.% RuNi SAA in hydrogenation of 4-nitrostyrene with the use of H_2 and D_2. Error bars were defined as the standard deviation by three replicate experiments.

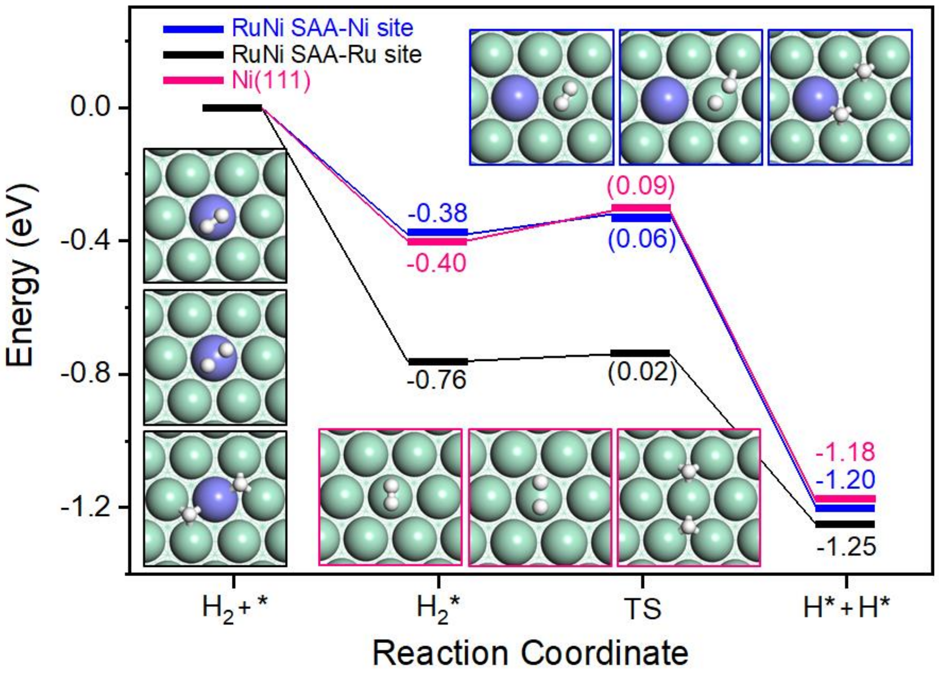

Supplementary Figure 35 | DFT studies for H_2 dissociation. Potential energy profiles for H_2 dissociation pathways on Ni(111) and RuNi SAA(111) surface, respectively. The DFT calculations of this figure were performed on a $p(5 \times 5)$ surface unit cell, and the Monkhorst-Pack k point mesh was set as $3 \times 3 \times 1$. Ru, violet; Ni, green; H, white.

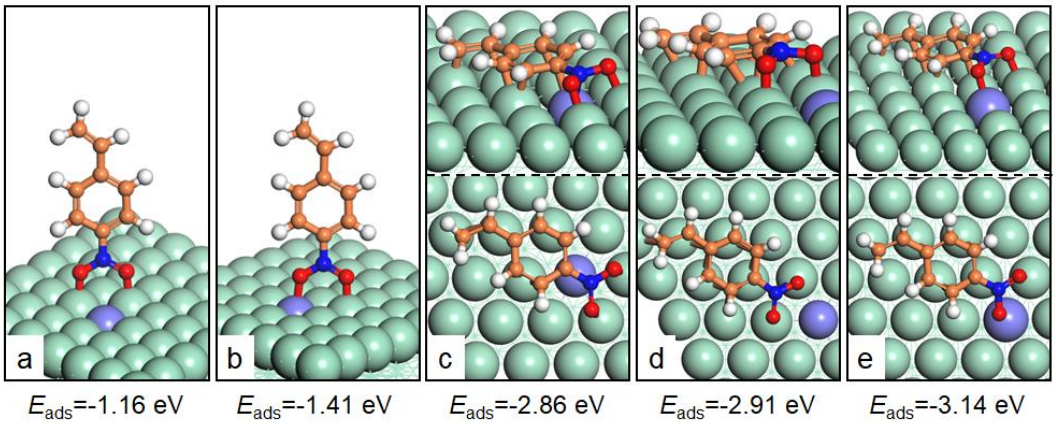

Supplementary Figure 36 | The adsorption behavior of 4-NS over RuNi SAA. a–e Proposed adsorption configurations and corresponding adsorption energies of 4-nitrostyrene on RuNi SAA(111) surface. Ru, violet; Ni, green; C, orange; O, red; N, blue; H, white.

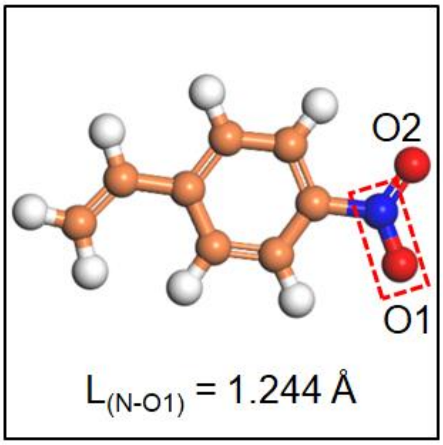

Supplementary Figure 37 | Structure drawing of 4-NS. Molecular structure of gaseous 4-nitrostyrene, with the labeled bond length of N−O1 ($L_{(N-O1)}$). C, orange; O, red; N, blue; H, white.

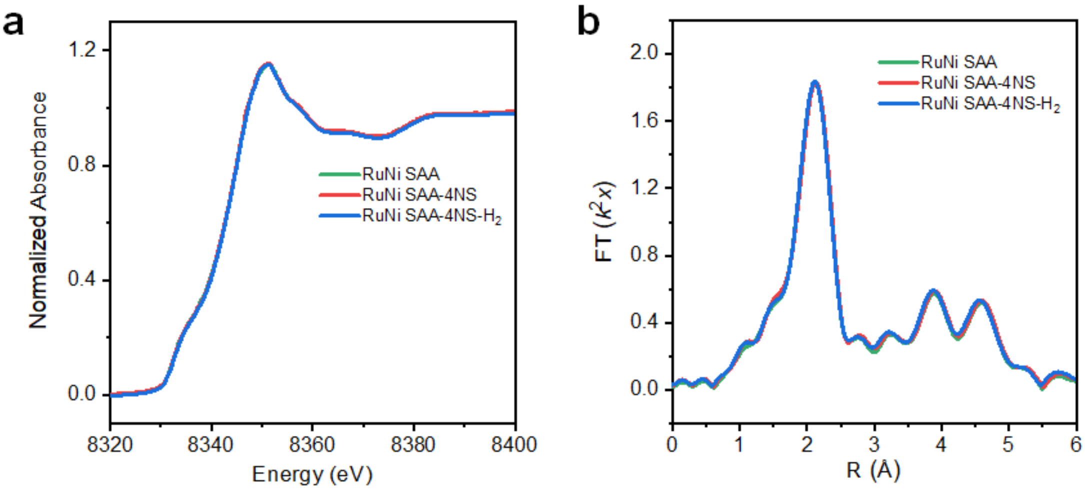

Supplementary Figure 38 | In situ XAFS studies of 4-NS adsorption and surface reaction. a In situ normalized XANES and **b** phase correction EXAFS Fourier–transform spectra at Ni K-edge for the 0.4 wt.% RuNi SAA sample in the cases of 4-NS adsorption (RuNi SAA−4NS), and hydrogenation reaction stage (RuNi SAA−4NS−H_2).

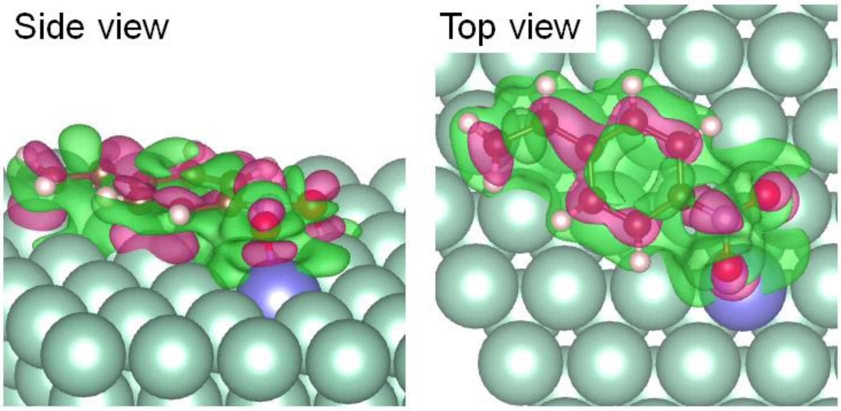

Supplementary Figure 39 | Charge density differential analysis. Charge density difference of 4-nitrostyrene adsorbed on the surface of RuNi SAA(111). The green and red contours represent electron accumulation and depletion, respectively. Ru, violet; Ni, green.

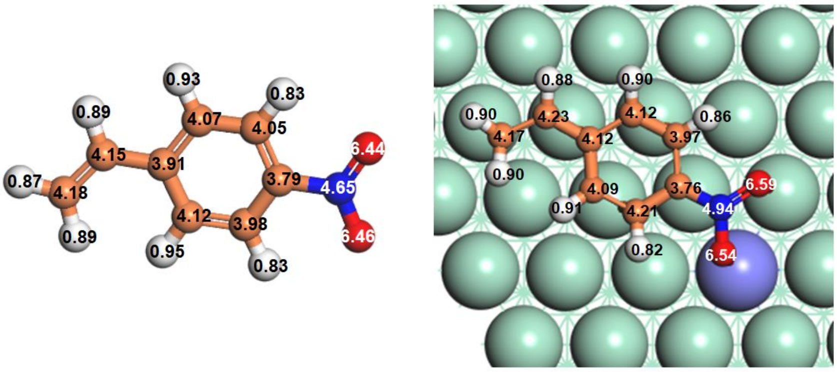

Supplementary Figure 40 | Bader charge analysis of 4-NS molecule. Bader charge of gaseous 4-nitrostyrene molecule and 4-nitrostyrene adsorbed on RuNi SAA(111) surface. The numbers represent the electron number carried by the outermost layer of atoms. Ru, violet; Ni, green; C, orange; O, red; N, blue; H, white.

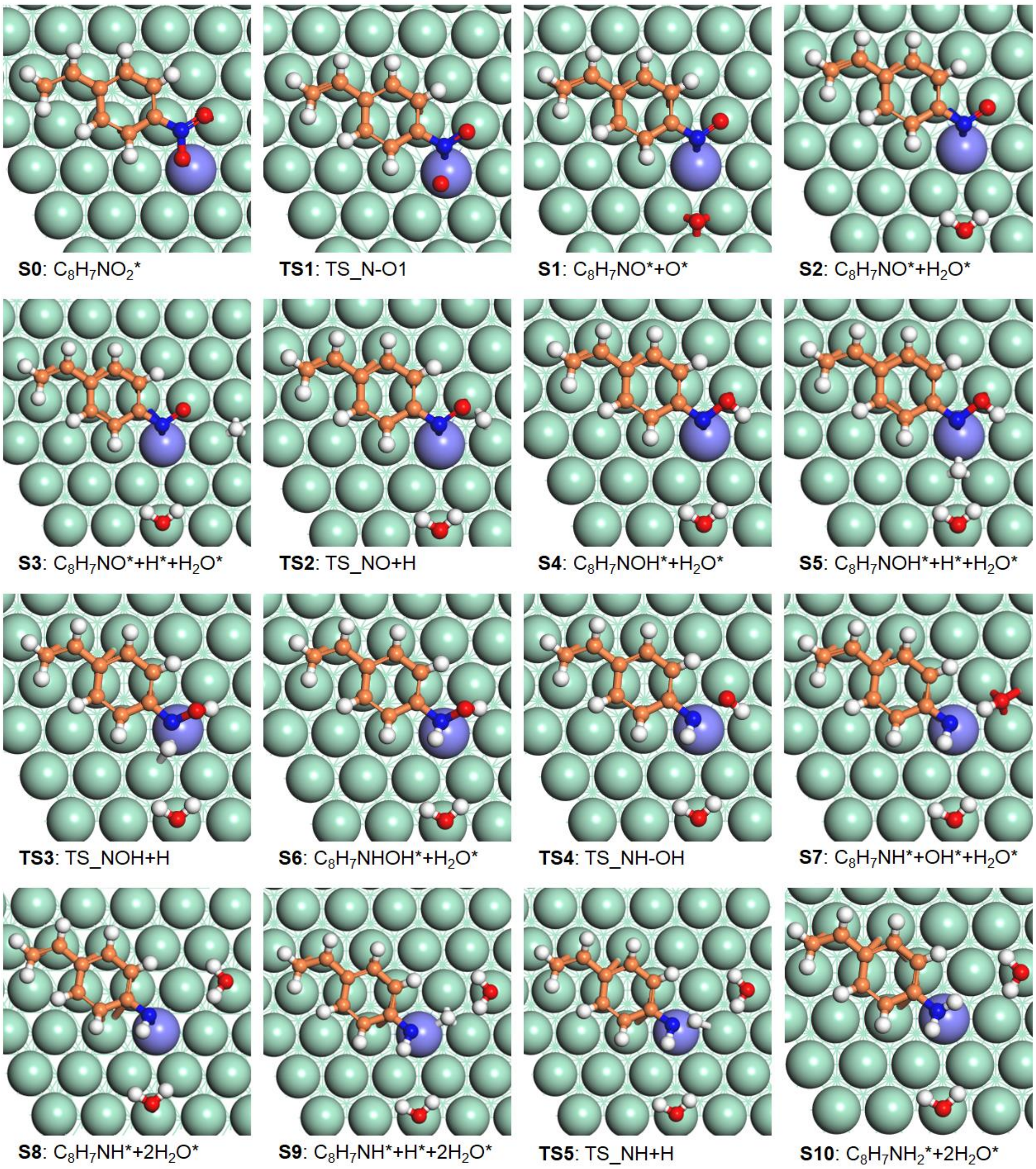

Supplementary Figure 41 | Optimized geometries of reaction intermediates. Geometric structures of the elementary step for 4-nitrostyrene hydrogenation to 4-aminostyrene on the surface of RuNi SAA(111). 'S0' denotes the initial state and 'S1−S10' represent a series of adsorption states. 'TS' denotes a transition state. Ru, violet; Ni, green; C, orange; O, red; N, blue; H, white.

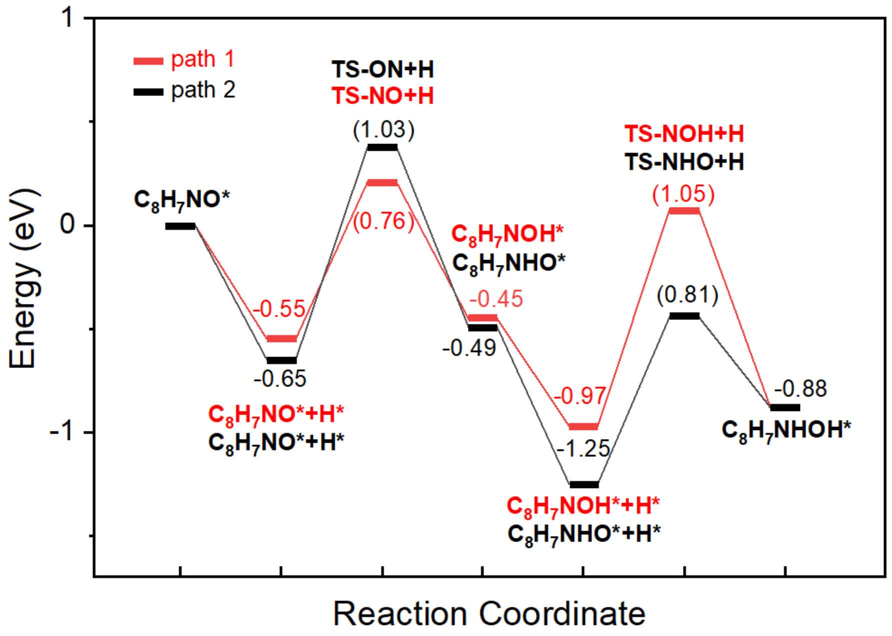

Supplementary Figure 42 | The exploration of reaction pathway. Potential energy profiles of proposed reaction pathway for the intermediate hydrogenation (from $C_8H_7NO^*$ to $C_8H_7NHOH^*$) over RuNi SAA(111). Path 1: active H atom preferentially attacks the O atom in $C_8H_7NO^*$; Path 2: active H atom hydrogenates N atom firstly. The hydrogenation energy barriers of $C_8H_7NO^*$ intermediate indicate that the active H atoms from Ru–Ni hollow sites preferentially attack O atom rather than N atom, giving rise to $C_8H_7NOH^*$. The DFT calculations of this figure were performed on a $p(5 \times 5)$ surface unit cell, and the Monkhorst-Pack k point mesh was set as $3 \times 3 \times 1$.

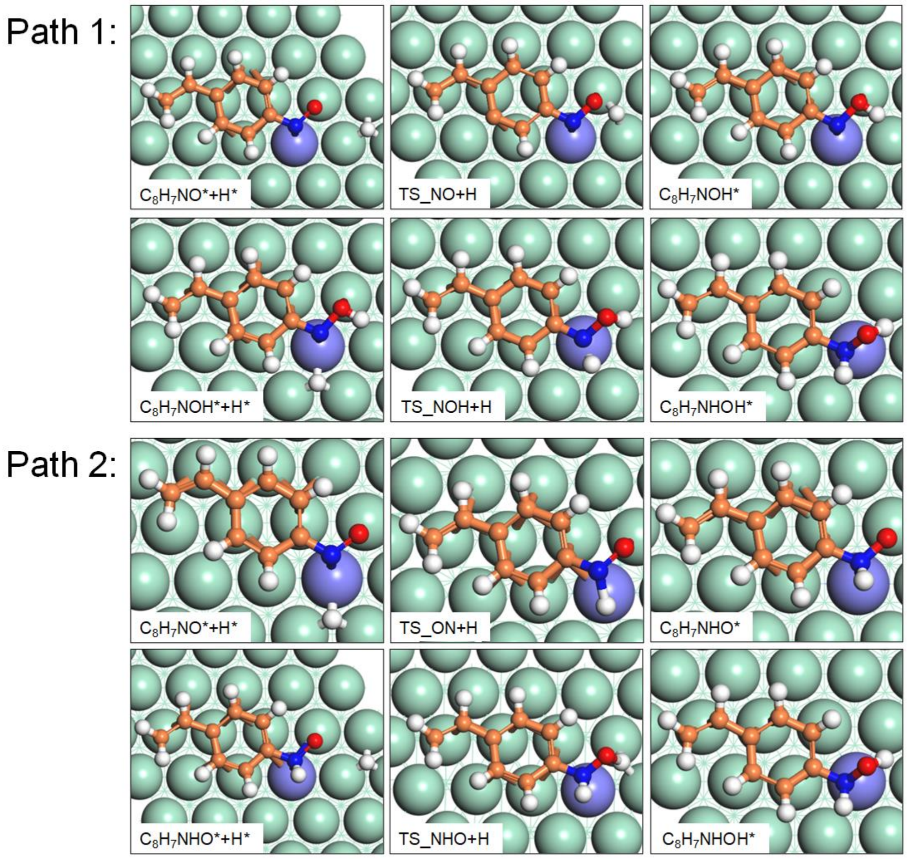

Supplementary Figure 43 | Geometries of intermediates for different reaction pathways. Geometric structures of the elementary step for the intermediate hydrogenation (from $C_8H_7NO^*$ to $C_8H_7NHOH^*$) via path 1 and path 2. Ru, violet; Ni, green; C, orange; O, red; N, blue; H, white.

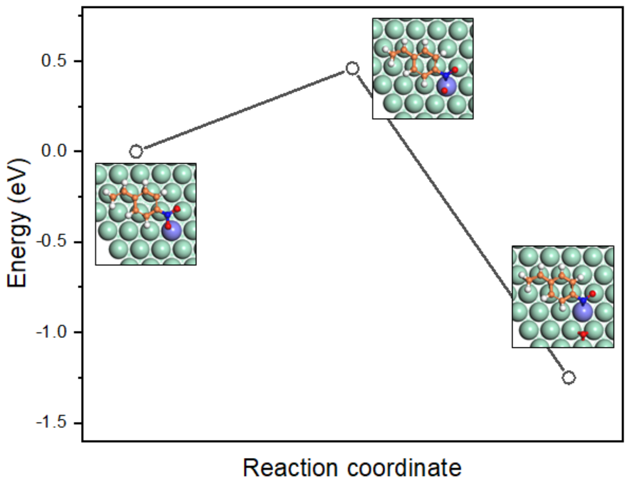

Supplementary Figure 44 | Energy curve of TS1. Energy curve with the reaction coordinates in $C_8H_7NO_2^* \rightarrow C_8H_7NO^* + O^*$ (TS1) on RuNi SAA.

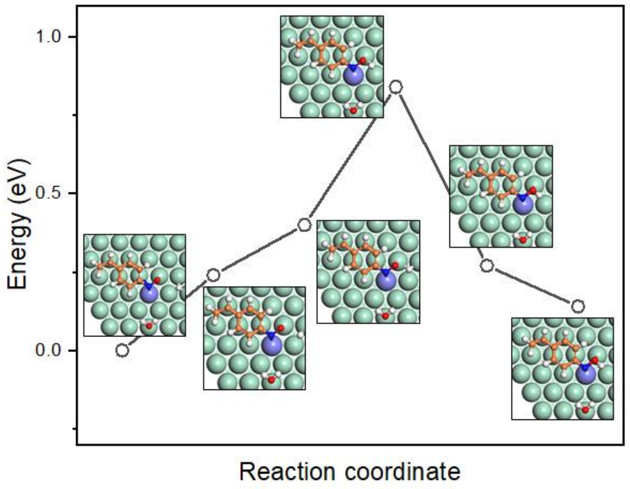

Supplementary Figure 45 | Energy curve of TS2. Energy curve with the reaction coordinates in $C_8H_7NO^* + H^* + H_2O^* \rightarrow C_8H_7NOH^* + H_2O^*$ (TS2) on RuNi SAA.

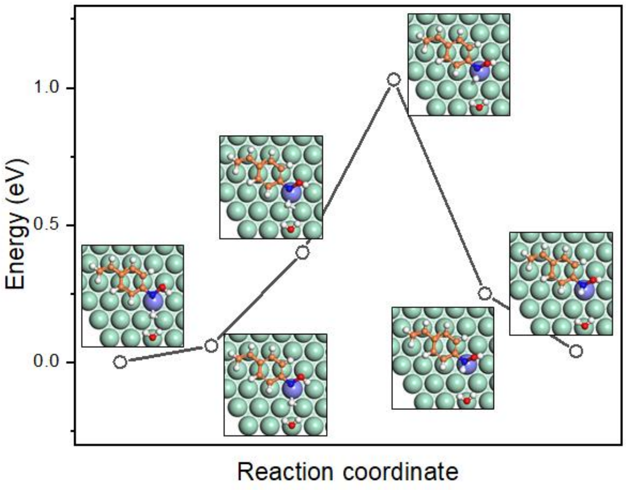

Supplementary Figure 46 | Energy curve of TS3. Energy curve with the reaction coordinates in $C_8H_7NOH^* + H^* + H_2O^* \rightarrow C_8H_7NHOH^* + H_2O^*$ (TS3) on RuNi SAA.

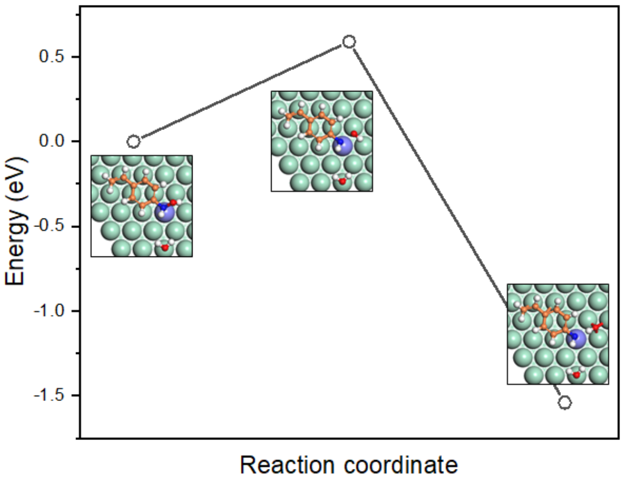

Supplementary Figure 47 | Energy curve of TS4. Energy curve with the reaction coordinates in $C_8H_7NHOH^* + H_2O^* \rightarrow C_8H_7NH^* + OH^* + H_2O^*$ (TS4) on RuNi SAA.

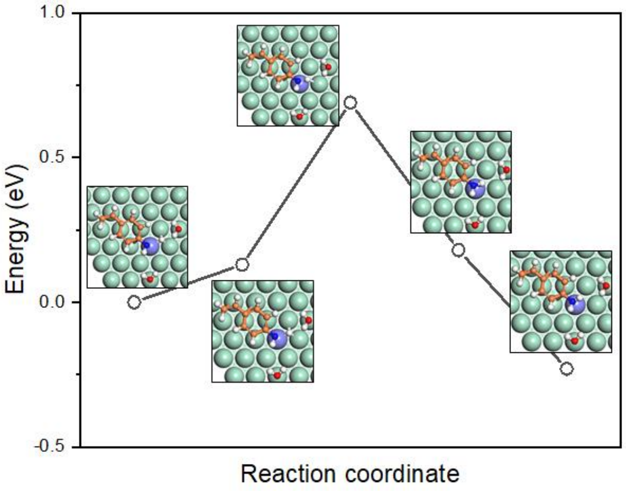

Supplementary Figure 48 | Energy curve of TS5. Energy curve with the reaction coordinates in $C_8H_7NH^* + H^* + 2H_2O^* \rightarrow C_8H_7NH_2^* + 2H_2O^*$ (TS5) on RuNi SAA.

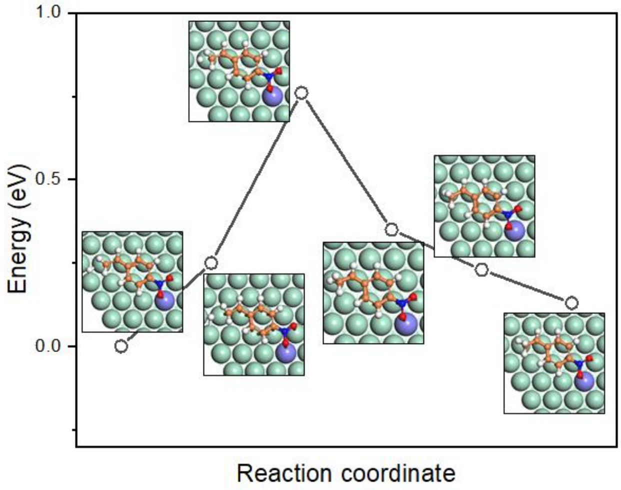

Supplementary Figure 49 | Energy curve of TS6. Energy curve with the reaction coordinates in $CH_2CH\text{-}C_6H_4NO_2^* + H^* \rightarrow CH_3CH\text{-}C_6H_4NO_2^*$ (TS6) on RuNi SAA.

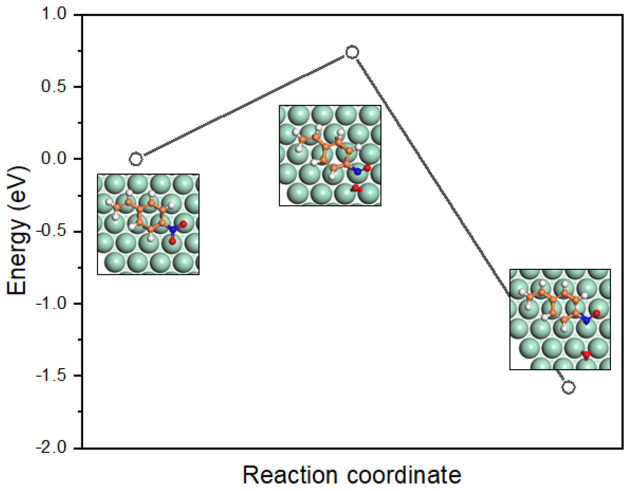

Supplementary Figure 50 | Energy curve of TS7. Energy curve with the reaction coordinates in $C_8H_7NO_2* \rightarrow C_8H_7NO* + O*$ (TS7) on Ni(111).

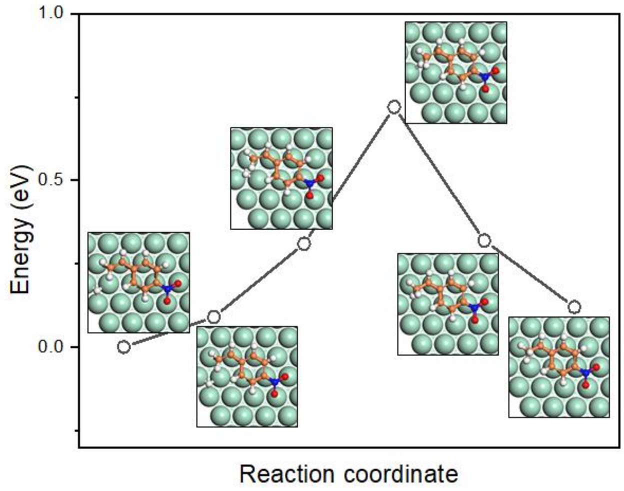

Supplementary Figure 51 | Energy curve of TS8. Energy curve with the reaction coordinates in $CH_2CH\text{-}C_6H_4NO_2* + H* \rightarrow CH_3CH\text{-}C_6H_4NO_2*$ (TS8) on Ni(111).

Supplementary Tables

Supplementary Table 1. Physicochemical parameters of various samples

Sample	BET surface area [a] ($m^2 \cdot g^{-1}$)	Mean pore size [a] (nm)	Ru species content [b] (wt.%)	Ni species content [b] (wt.%)	Mean metal particle size [c] (nm)	Surface metal atoms content [d] ($mmol \cdot g^{-1}$)
Ni/Al_2O_3	182.5	6.2	–	23.8	7.9	0.386
0.1% RuNi	159.0	6.5	0.07	24.4	7.7	0.398
0.2% RuNi	134.3	7.6	0.16	23.2	8.3	0.389
0.4% RuNi	141.6	8.3	0.29	23.5	8.0	0.402
0.6% RuNi	150.9	7.5	0.45	22.7	7.9	0.395
0.8% RuNi	139.3	7.9	0.61	23.0	8.2	0.420
1% RuNi	119.8	7.5	0.73	22.6	7.8	0.415
2% RuNi	116.9	8.1	1.52	22.1	8.1	0.457

[a] Specific surface area and mean pore size were determined by BET measurements. [b] Contents of metal Ru and Ni were measured by ICP–AES. [c] Mean metal particle size of Ni was determined by TEM images. [d] Surface metal atoms content was calculated based on the results of CO pulse chemisorption.

Supplementary Table 2. Catalytic performance of Al_2O_3, 0.4 wt.% Ru/Al_2O_3 and 2 wt.% Ru/Al_2O_3 samples toward selective hydrogenation of 4-nitrostyrene

Entry	Catalyst	Time (h)	Tem. (°C)	P_{H2} (MPa)	Con. (%)	Sel. (%) 4-AS	4-NE	4-AE
1	Al_2O_3	3	60	1	0.6	/	/	/
2	0.4% Ru/Al_2O_3	3	60	1	12.9	67.5	24.7	7.8
3	2% Ru/Al_2O_3	3	60	1	100	15.7	52.9	31.4

Reaction conditions: 1 mmol of reactant (4-NS); 8 ml of solvent (ethanol); 0.02 g of catalyst; 1 Mpa of H_2, 60 °C, 3 h.

Supplementary Table 3. Comparison results on catalytic performance toward 4-nitrostyrene hydrogenation to 4-aminostyrene

Entry	Catalyst	Time (h)	Tem. (°C)	P_{H2} (MPa)	Con. (%)	Sel. (%)	TOF (h^{-1})	Reference
1	0.1% RuNi SAA	3	60	1	100	>99	4287 [a]<br>2143 [b]<br>74.6 [c]	This work
2	0.2% RuNi SAA	3	60	1	100	>99	4271 [a]<br>2135 [b]<br>173.7 [c]	This work
3	0.4% RuNi SAA	3	60	1	100	>99	4293 [a]<br>2146 [b]<br>306.3 [c]	This work
4	Pd/Ti_3SiC_2	24	140	2.5	100	93	4700	19
5	PtTW1	0.06	80	1	80	66	1880	20
6	$Co_{0.23}$-$Ru_{0.77}$	12	25	0.3	97	99	32	21
7	$RhIn/SiO_2$	2	75	0.1	99	93	25	22
8	Pt@MFI	13	80	2	100	83	16	23
9	Au/TiO_2/UVM-7	24	140	2.5	30	94	3.3	24
10	Co/N-C	3	100	1	>99	97	1.9	25
11	Ni-Fe/SiO_2	4.5	70	0.1	98	95	11	26
12	Ir_1Mo_1/TiO_2	1	120	2	100	>96	–	27
13	Pd nanosheets	0.1	25	1	100	0	–	28
14 [d]	$Pd_{13}Pb_9$	1	90	–	99	96	–	29
15 [e]	Pt_1/Fe_2O_3	0.3	60	0.3	98.6	98	3809	30
16 [e]	Pt_1/Ni	2	40	0.3	97	99	1800	31
17 [e]	0.08%Pt/FeO_x	0.83	40	0.3	96.5	98.6	1514	32
18 [e]	Co-N-C	2	80	1	80.3	97.8	9.6	33

[a-c] The TOF values were calculated as the molar amount of 4-NS converted per mole of metal sites (including the Ru sites [a], the Ru–Ni interfacial sites [b] and the total surface metal atoms [c]) per hour. [d] A catalytic transfer hydrogenation reaction without using H_2. [e] 3-nitrostyrene was used as reactant.

Supplementary Table 4. Catalytic performance toward chemoselective hydrogenation of various nitroarenes over 0.4% RuNi SAA catalyst

Entry	Reactant	Product	Con. (%)	Sel. (%)
1	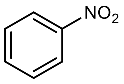	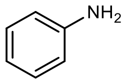	100	100
2	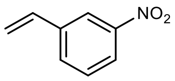	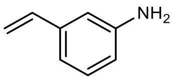	100	98
3	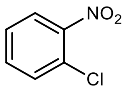	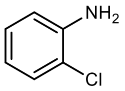	100	99
4	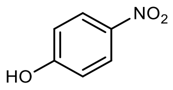	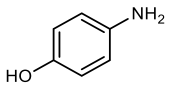	99	96
5	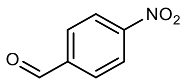	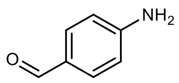	100	95

Reaction conditions: 1 mmol of reactant; 8 ml of solvent (ethanol); 0.02 g of catalyst (0.4% RuNi SAA); 1 Mpa of H_2, 60 °C, 3 h.

Supplementary Table 5. Curve-fitting results of Ru K-edge EXAFS spectra for various samples

**Sample**	**Shell**	**R (Å)** [a]	**CN** [b]	σ^2 **(10^{-3} Å^2)** [c]	ΔE_0 **(eV)** [d]	***R* factor (%)**
Ru-foil	Ru–Ru	2.67(±0.01)	12	3.5	2.27	0.8
RuO_2	Ru–O	1.97(±0.01)	6	2.3	3.72	0.7
0.4% RuNi	Ru–Ni	2.47(±0.01)	5.4	7.4	3.90	0.5
0.6% RuNi	Ru–Ni	2.52(±0.01)	5.8	6.8	8.50	0.7
	Ru–Ru	2.62(±0.02)	2.2	5.5		
2% RuNi	Ru–Ni	2.47(±0.04)	2.5	8.0	4.41	0.8
	Ru–Ru	2.65(±0.01)	6.7	4.2		
Ru/Al_2O_3	Ru–Ru	2.63(±0.01)	6.2	5.6	2.52	1.2
	Ru–O	2.07(±0.1)	2.9	9.2		

[a] R: bond distance; [b] CN: coordination number; [c] σ^2: Debye-Waller factor; [d] ΔE_0: the inner potential correction. The accuracies of the above parameters were estimated as follows: CN, ±20%; R, ±1%; σ^2, ±20%; ΔE_0, ± 20%. The data ranges used for data fitting in k space (Δk) and R space (ΔR) are 2.5−12.0 Å^{-1} and 1.0−3.0 Å, respectively.

Supplementary Table 6. Lattice parameters of bulk Ni calculated with four exchange-correlation functions, in comparison with experimental value

**Method**	**Lattice constant (Å)**
PBE-D3	3.524
PBEsol	3.471
PBE	3.516
PW91	3.524
Expt.[34]	3.524

Supplementary References:

1. Ravel, B. & Newville, M. ATHENA, ARTEMIS, HEPHAESTUS: data analysis for X-ray absorption spectroscopy using IFEFFIT. *J. Synchrotron Radiat.* **12**, 537−541 (2005).

2. Funke, H., Chukalina, M. & Scheinost, A. C. A new FEFF-based wavelet for EXAFS data analysis. *J. Synchrotron Radiat.* **14**, 426−432 (2007).

3. Kresse, G. & Furthmüller, J. Efficiency of ab-initio total energy calculations for metals and semiconductors using a plane-wave basis set. *Comput. Mater. Sci*. **6**, 15−50 (1996).

4. Kresse, G. & Furthmüller, J. Efficient iterative schemes for ab initio total-energy calculations using a plane-wave basis set. *Phys. Rev. B: Condens. Matter Mater. Phys*. **54**, 11169−11186 (1996).

5. Blöchl, P. E. Projector augmented-wave method. *Phys. Rev. B: Condens. Matter Mater. Phys*. **50**, 17953−17979 (1994).

6. Kresse, G. & Joubert, D. From ultrasoft pseudopotentials to the projector augmented-wave method. *Phys. Rev. B: Condens. Matter Mater. Phys*. **59**, 1758−1775 (1999).

7. Perdew, J. P., Burke, K. & Ernzerhof, M. Generalized gradient approximation made simple. *Phys. Rev. Lett*. **77**, 3865−3868 (1996).

8. Grimme, S., Antony, J., Ehrlich, S. & Krieg, H. A consistent and accurate ab initio parametrization of density functional dispersion correction (DFT-D) for the 94 elements H-Pu. *J. Chem. Phys.* **132**, 154104 (2010).

9. Perdew, J. P. et al. Restoring the density-gradient expansion for exchange in solids and surfaces. *Phys. Rev. Lett*. **100**, 136406 (2008).

10. Delley, B. An all-electron numerical method for solving the local density functional for polyatomic molecules. *J. Chem. Phys*. **92**, 508−517 (1990).

11. Perdew, J. P. et al. Atoms, molecules, solids, and surface: applications of the generalized gradient approximation for exchange and correlation. *Phys. Rev. B: Condens. Matter Mater. Phys*. **46**, 6671−6687 (1992).

12. Chen, L. et al. Catalytic conversion furfuryl alcohol to tetrahydrofurfuryl alcohol and 2-methylfuran at terrace, step, and corner sites on Ni. *ACS Catal*. **10**, 7240−7249 (2020).

13. Yang, J., Lv, C., Guo, Y. & Wang, G. A DFT+U study of acetylene selective hydrogenation on oxygen defective anatase (101) and rutile (110) TiO_2 supported Pd_4 cluster. *J. Chem. Phys.* **136**, 104107 (2012).

14. Heyden, A., Bell, A. T. & Keil, F. J. Efficient methods for finding transition states in chemical reactions: Comparison of improved dimer method and partitioned rational function optimization method. *J. Chem. Phys*. **123**, 224101 (2005).

15. Henkelman, G., Arnaldsson, A. & Jónsson, H. A fast and robust algorithm for Bader decomposition of charge density. *Comput. Mater. Sci*. **36**, 354−360 (2006).

16. Sanville, E., Kenny, S. D., Smith, R. & Henkelman, G. Improved grid-based algorithm for Bader charge allocation. *J. Comput. Chem*. **28**, 899−908 (2007).

17. Tang, W., Sanville, E. & Henkelman, G. A grid-based Bader analysis algorithm without lattice bias. *J. Phys.: Condens. Matter*. **21**, 084204 (2009).

18. Momma, K. & Izumi, F. VESTA 3 for three-dimensional visualization of crystal, volumetric and morphology data. *J. Appl. Crystallogr.* **44**, 1272−1276 (2011).

19. Trandafir, M. M. et al. Highly efficient ultralow Pd loading supported on MAX phases for chemoselective hydrogenation. *ACS Catal.* **10**, 5899−5908 (2020).

20. Carrus, M. et al. Increased conversion and selectivity of 4-nitrostyrene hydrogenation to 4-aminostyrene on Pt nanoparticles supported on titanium-tungsten mixed oxides. *Appl. Catal. A* **519**, 130−138 (2016).

21. Mao, J. et al. Rational control of the selectivity of a ruthenium catalyst for hydrogenation of 4-nitrostyrene by strain regulation. *Angew. Chem. Int. Ed.* **56**, 11971−11975 (2017).

22. Furukawa, S., Takahashi, K. & Komatsu, T. Well-structured bimetallic surface capable of molecular recognition for chemoselective nitroarene hydrogenation. *Chem. Sci.* **7**, 4476−4484 (2016).

23. Gu, J. et al. Platinum nanoparticles encapsulated in MFI zeolite crystals by a two-step dry gel conversion method as a highly selective hydrogenation catalyst. *ACS Catal.* **5**, 6893−6901 (2015).

24. Trandafir, M. M., Moragues, A., Amorós, P. & Parvulescu, V. I. Selective hydrogenation of nitroderivatives over Au/TiO_2/UVM-7 composite catalyst. *Catal. Today* **355**, 893−902 (2020).

25. Wang, X. & Li, Y. Chemoselective hydrogenation of functionalized nitroarenes using MOF-derived Co-based catalysts. *J. Mol. Catal. A-Chem.* **420**, 56−65(2016).

26. Miyazaki, M., Ariyama, K., Furukawa, S., Takayama, T. & Komatsu, T. Chemoselective hydrogenation of nitroarenes using Ni-Fe alloy catalysts at ambient pressure. *ChemistrySelect* **6**, 5538−5544 (2021).

27. Fu, J. et al. Synergistic effects for enhanced catalysis in a dual single-atom catalyst. *ACS Catal.* **11**, 1952−1961 (2021).

28. Yang, N. et al. Amorphous/crystalline hetero-phase Pd nanosheets: one-pot synthesis and highly selective hydrogenation reaction. *Adv. Mater.* **30**, 1803234 (2018).

29. Furukawa, S., Yoshida, Y. & Komatsu, T. Chemoselective hydrogenation of nitrostyrene to aminostyrene over Pd- and Rh-based intermetallic compounds. *ACS Catal.* **4**, 1441−1450 (2014).

30. Ren, Y. et al. Unraveling the coordination structure-performance relationship in Pt_1/Fe_2O_3 single-atom catalyst. *Nat. Commun.* **10**, 4500 (2019).

31. Peng, Y. et al. Pt single atoms embedded in the surface of Ni nanocrystals as highly active catalysts for selective hydrogenation of nitro compounds. *Nano Lett.* **18**, 3785−3791 (2018).

32. Wei, H. et al. FeO_x-supported platinum single-atom and pseudo-single-atom catalysts for chemoselective hydrogenation of functionalized nitroarenes. *Nat. Commun.* **5**, 5634 (2014).

33. Li, M. et al. Origin of the activity of Co-N-C catalysts for chemoselective hydrogenation of nitroarenes. *ACS Catal.* **11**, 3026−3039 (2021).

34. Suh, I. K., Ohta, H. & Waseda, Y. High-temperature thermal expansion of six metallic elements measured by dilatation method and X-ray diffraction. *J. Mater. Sci.* **23**, 757−760 (1988).
